# The Asian Rice Gall Midge (*Orseolia oryzae*) Mitogenome Has Evolved Novel Gene Boundaries and Tandem Repeats That Distinguish Its Biotypes

**DOI:** 10.1371/journal.pone.0134625

**Published:** 2015-07-30

**Authors:** Isha Atray, Jagadish Sanmallappa Bentur, Suresh Nair

**Affiliations:** 1 Plant Molecular Biology Group, International Centre for Genetic Engineering and Biotechnology, New Delhi, 110067, India; 2 Directorate of Rice Research, Rajendranagar, Hyderabad, India; Sichuan University, CHINA

## Abstract

The complete mitochondrial genome of the Asian rice gall midge, *Orseolia oryzae* (Diptera; Cecidomyiidae) was sequenced, annotated and analysed in the present study. The circular genome is 15,286 bp with 13 protein-coding genes, 22 tRNAs and 2 ribosomal RNA genes, and a 578 bp non-coding control region. All protein coding genes used conventional start codons and terminated with a complete stop codon. The genome presented many unusual features: (1) rearrangement in the order of tRNAs as well as protein coding genes; (2) truncation and unusual secondary structures of tRNAs; (3) presence of two different repeat elements in separate non-coding regions; (4) presence of one pseudo-tRNA gene; (5) inversion of the rRNA genes; (6) higher percentage of non-coding regions when compared with other insect mitogenomes. Rearrangements of the tRNAs and protein coding genes are explained on the basis of tandem duplication and random loss model and why intramitochondrial recombination is a better model for explaining rearrangements in the *O*. *oryzae* mitochondrial genome is discussed. Furthermore, we evaluated the number of iterations of the tandem repeat elements found in the mitogenome. This led to the identification of genetic markers capable of differentiating rice gall midge biotypes and the two *Orseolia* species investigated.

## Introduction

The Asian rice gall midge, *Orseolia oryzae* (Diptera: Cecidomyiidae) is an important pest of rice that is responsible for an average yield loss of US $ 80 million annually in India alone [1]. Cultural, biological and chemical control methods have been employed to alleviate this loss. The most effective method of controlling the pest has been the development of resistant rice varieties whose extensive cultivation could be responsible for the emergence of new virulent biotypes and breakdown of host resistance genes [2,3]. To get a better understanding of the molecular basis of gall midge-rice interaction, a thorough knowledge of the genetic constitution of the insect and the plant is a pre-requisite. While rice genes/genetic pathways involved in the interactions are being investigated [4,5,6] such information is currently lacking as far as the rice gall midge is concerned. As resistance to gall midge is primarily due to antibiosis and specific to the plant and insect genotypes involved, it is essential to study population genetic structure of *O*. *oryzae*. Analysis of the mitochondrial genome of the Asian rice gall midge would help address this lacuna and in generating molecular markers to track genome evolution and insect phylogeny [7,8].

The mitochondrial genome is special because of its small size, maternal inheritance, the lack of recombination and the presence of sequence polymorphisms between different taxa. This makes the mitochondrial genome very useful while analysing molecular evolution and carrying out population and phylogenetic studies in arthropods [9,10,11]. The insect mitochondrial genome is usually a circular, double-stranded DNA molecule of approximately 14–16 kb. The genome has a total of 37 genes comprising of 13 protein-coding genes (PCGs), 22 tRNAs and 2 rRNA genes. Besides these, it also has a large non-coding region, the control region, which harbours elements responsible for regulating replication and transcription of mitochondrial genomes.

Gene arrangements in mitochondrial genomes are considered to be informative in evolutionary studies and generally it has been observed that the rearrangements in mitogenomes are restricted to tRNA genes. However, studies have also shown that three of the four orders of the hemipteroid complex (Phthiraptera, Psocoptera and Thysanoptera) show rearrangements of tRNAs as well as the PCGs compared to the ancestral order. However, species belonging to Hemiptera (the fourth order of the hemipteroid complex) maintain the ancestral gene order. In contrast, rearrangements in Diptera are restricted to tRNAs such as inversion of *trnS1* in Culicidae [12,13] and the inversion of *trnT* and *trnP* without any translocation as observed in other gall midges from the family Cecidomyiidae [14].

In this study, we present the complete sequence of the mitochondrial genome of the Asian rice gall midge, *O*. *oryzae* highlighting its unique features such as highly rearranged gene order, involving both PCGs and tRNAs; tandem repeats at two separate non-coding regions which have the potential to differentiate gall midge biotypes and species of the genus and truncation in the predicted secondary structures of the tRNAs. These features are also compared with those known for other insect mitogenomes.

## Materials and Methods

### Collection of *Orseolia* species and DNA isolation

The Asian rice gall midge, *Orseolia oryzae*, biotype 1 (GMB1) insects were collected from the Directorate of Rice Research (DRR), Hyderabad, India and kept in 100% ethanol at -20°C for long term storage at the International Centre for Genetic Engineering and Biotechnology (ICGEB), until used for DNA isolation. Adult insects of GMB2, GMB3, GMB4, GMB4M, GMB5 and GMB6 and *O*. *fluvialis* were supplied by DRR and preserved in a similar fashion. The DNA from the African rice gall midge (*O*. *oryzivora*) was available from a previous study [15]. DNA was isolated by crushing the insects using a micropestle in extraction buffer (1% SDS, 0.05 M NaCl; 0.05 M Tris-HCl, pH 8.0; 0.025 M EDTA). This was then followed by an RNase and a Proteinase K treatment. Next, the DNA was extracted once with phenol:chloroform:isoamyl alcohol (25:24:1) and then with chloroform:isoamyl alcohol (24:1). DNA was finally precipitated with ethanol and resuspended in distilled water and quantified using NanoVue (GE Healthcare, UK).

### PCR, cloning and sequencing

The mitogenome was amplified using PCR in overlapping fragments using primers mentioned in Simon et al. [16], and subsequent primers designed from sequenced fragments in the present study (S1 Table). The PCRs were performed with four different Taq polymerase enzymes: Taq DNA polymerase (Promega), Taq DNA polymerase (Bangalore Genei), Kapa 2G Robust (Kapa Biosystems) and Prime Star GXL Taq DNA polymerase (Takara Bio. Inc.). A total of 18 overlapping fragments were generated using the following conditions: 2 minutes initial denaturation at 94°C, followed by 30 cycles of 30s at 94°C, 1 min at 41–55°C and 1–2 min at 65°C and a final elongation at 65°C for 2 mins. The PCR products were electrophoresed in a 0.8% agarose gel and purified using the Gel extraction kit (Qiagen, Catalogue no. 28704). The gel extracted PCR products were cloned in the pCR4-TOPO vector using the TOPO-TA cloning kit (Invitrogen, Catalogue no. 450030) and transformed into DH5α competent cells. The plasmids were isolated using the Qiaprep Spin Miniprep kit (Qiagen, Catalogue no. 27104). All fragments were sequenced using the Big Dye capillary sequencing method (Macrogen, South Korea).

### Analysis of nucleotide sequences

The sequencing data were analysed and assembled into contigs using the MacVector software (version 13.0.3). The protein coding genes (PCGs) were identified by BLASTX (http://blast.ncbi.nlm.nih.gov/Blast) searches and using the invertebrate mitochondrial genetic code. The PCGs and the ribosomal RNA genes were compared with other homologous insect mitochondrial sequences using MacVector to ensure their accurate boundaries. The transfer RNAs were predicted using the DOGMA software (http://dogma.ccbb.utexas.edu/) and also tRNAscan-SE using COVE threshold as 1. The nucleotide composition and codon usage were analysed using MEGA (version 5.2) [17]. The secondary structure of the putative stem loop of the control region was folded using Mfold (http://mfold.rit.albany.edu/) [18]. The mechanism for gene order rearrangement was deduced using the CREx (Common Interval Rearrangement Explorer) online tool (http://pacosy.informatik.uni-leipzig.de/crex) [19]. The repeats in the fragments were determined using the Tandem Repeats Finder online tool (http://tandem.bu.edu/trf/trf.html) [20]. Strand asymmetry was calculated using the formulae: AT skew = [A-T]/[A+T] and GC skew = [G-C]/[G+C].

### Phylogenetic analysis

Phylogenetic analysis was performed with 25 complete mitochondrial genomes of other arthropods (S2 Table). Genome sequences from nine different arthropod orders were taken for the construction of the phylogenetic tree. The sequences were downloaded from GenBank and aligned using CLUSTAL W in the MacVector suite of programs. This sequence alignment file was then used to construct phylogenetic tree using the Maximum Likelihood method in MEGA.

## Results and Discussion

### Genome organization and structure

**Fig 1:**
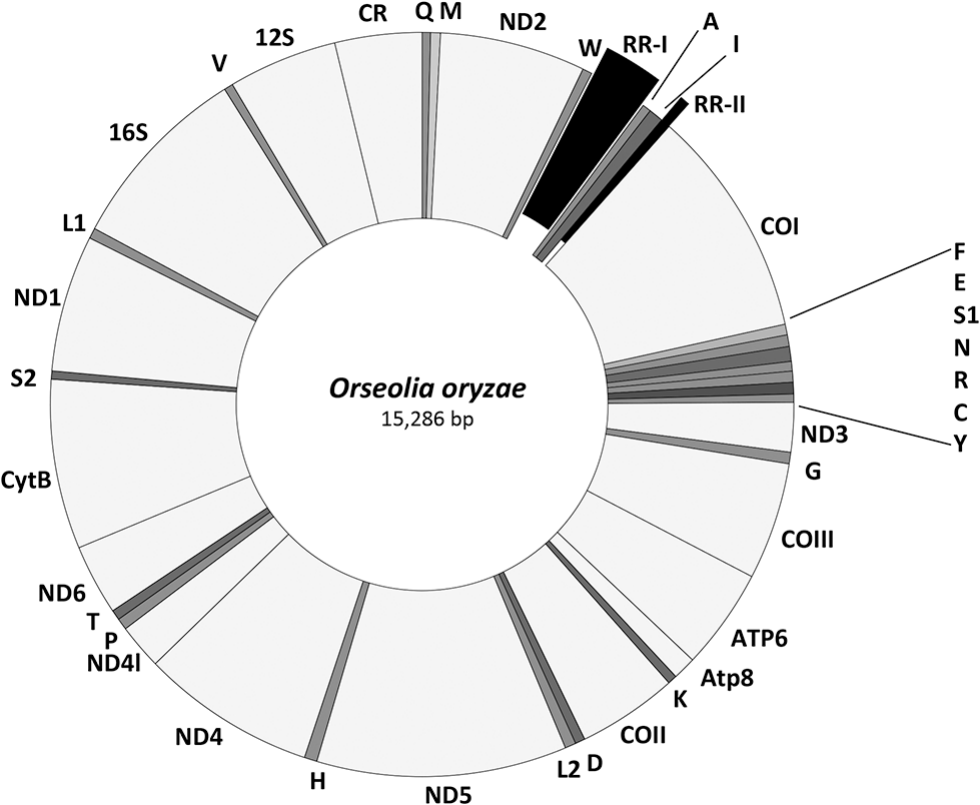
The structure of *Orseolia oryzae* mitochondrial genome. The light grey areas indicate the PCGs, rRNAs and the control region and the dark grey areas indicate the different tRNAs. The exploding slices of the doughnut indicate the location of two repeat regions, RR-I and RR-II.

The complete mitochondrial genome of *O*. *oryzae* is a circular, double stranded DNA molecule of 15,286 bp (Fig 1). The mitogenome was assembled using 18 overlapping cloned PCR fragments. These fragments were 400 bp to 2.0 Kb long enabling large overlaps at the two ends of these fragments. This further ensured that the assembly did not contain nuclear sequences of mitochondrial origin (numts). Two clones of each fragment were sequenced and contigs were generated. The mitogenome has the typical number of 37 genes, 13 PCGs, 2 rRNAs and 22 tRNAs (Table 1), as reported for other insects. In addition, a 578 bp control region (CR) was present between 12S rRNA and trn*Q*. A distinctive feature identified in the mitogenome of *O*. *oryzae* was the presence of two repeat regions, RR-I and RR-II located between trn*W* and trn*A* and between trn*I* and COI, respectively. Such repeats have been previously unreported from any other arthropod mitogenomes. In addition to the control region and the repeat regions, 665 nucleotides of non-coding DNA was present dispersed in the genome. Among the different intergenic spacer regions found between various genes, the longest was 155 bp between trn*I* and COI and the smallest 1 bp between COIII and ATP6. The order of the genes was found to be different from the conserved arthropod gene organization. Twenty genes were either translocated and/or inverted, out of which 15 were tRNAs and 5 were PCGs. In all, 15 gene boundaries shared overlapping sequences that ranged from 2 (between trn*T* and ND6) to 31 nucleotides (between ND6 and trn*H*).

**Table 1:** Annotation and gene organization of the *Orseolia oryzae* mitogenome.

Feature	Strand	Size (bp)	Anticodon	Start Codon	Stop Codon	Length of variable arm (bp)
***trn*Q**	J	56	TTG	-	-	3
***trn*M**	N	65	CAT	-	-	9
**ND2**	N	969	-	ATT	TAA	-
***trn*W**	N	64	TCA	-	-	Replaced by TV loop
***trn*A**	N	58	TGC	-	-	Replaced by TV loop
***trn*I**	N	67	AAT	-	-	5
**COI**	N	1539	-	ATT	TAA	-
***trn*F**	N	71	GAA	-	-	5
***trn*E**	J	77	TTC	-	-	8
***trn*S1**	J	98	TCT	-	-	4
***trn*N**	N	64	ATT	-	-	6
***trn*R**	J	73	TCG	-	-	6
***trn*C**	N	77	GCA	-	-	4
***trn*Y**	J	58	ATA	-	-	0
**ND3**	J	342	-	ATT	TAA	-
***trn*G**	J	77	TCC	-	-	6
**COIII**	J	780	-	ATT	TAA	-
**ATP6**	J	678	-	-	-	-
**ATP8**	J	147	-	-	-	-
***trn*K**	J	61	TTT	-	-	10
**COII**	J	669	-	ATT	TAA	-
***trn*D**	J	66	GTC	-	-	5
***trn*L2**	J	73	TAA	-	-	15
**ND5**	J	1698	-	ATA	TAA	-
***trn*H**	J	85	GTG	-	-	18
**ND4**	J	1185	-	ATA	TAA	-
**ND4L**	J	300	-	ATT	TAA	-
***trn*P**	J	79	TGT	-	-	8
***trn*T**	N	68	TGG	-	-	0
**ND6**	N	456	-	ATA	TAA	-
**CytB**	N	1128	-	ATT	TAA	-
***trn*S2**	N	54	TGA	-	-	9
**ND1**	J	903	-	ATA	TAA	-
***trn*L1**	J	70	TAA	-	-	5
**16 S**	J	1262	-	-	-	-
***trn*V**	J	60	TAC	-	-	4
**12 S**	J	726	-	-	-	-

(J: Major strand, N: Minor Strand)

### Protein coding genes (PCGs)

Using BLASTX searches, all the 13 PCGs could be identified in the *O*. *oryzae* mitochondrial genome (Table 1). All of them initiated with an ATN start codon (one with ATG, seven with ATT and five with ATA) and terminated with a complete stop codon (twelve with TAA and one with TAG), which is in contrast to other insect mitogenomes where genes commonly terminate with incomplete stop codons such as T or TA. It is presumed that the TAA stop codon is restored by the addition of 3’ A-residues as a result of post-transcriptional polyadenylation [21].

The start and stop codons of all the PCGs were compared across different insects in Diptera (S3 Table). It was found that ATG was the start codon initiating ATP6 in all insects. ATC was used as a start codon only in the mosquitoes where it was used to initiate ND2 and ND5 in *Culex quinquefasciatus*, ND2 and ATP8 in *Anopheles gambiae* and ND5 in *Aedes aegypti*. TTG initiated translation only in *Mayetiola destructor* for ND4L. Out of all the insects compared, only *M*. *destructor* and *O*. *oryzae* showed termination of all genes with complete stop codons. Also, TAG codon terminated ND4 in *M*. *destructor* and ND6 in *O*. *oryzae*. It has been observed that the start codon for the COI gene is non-traditional in the case of Dipterans and Hemipterans [13]. However, in *O*. *oryzae* it is initiated with a traditional ATT start codon conforming to the invertebrate mitochondrial genetic code. Thus, it was observed that the start and stop codons in the *O*. *oryzae* mitogenome are different from those reported for other dipterans.

A comparison of the lengths of the different PCGs in dipterans revealed that gene sizes in *O*. *oryzae* were smaller in most of the cases compared with those from other dipteran members and in contrast, the non-coding regions in *O*. *oryzae* mitogenome were significantly longer (S1 File). This could be a result of the evolutionary pressures on the mitogenome to retain a smaller size.

The mitogenome of insects has two strands that can be differentiated into the major strand and the minor strand. The strand that codes for most of the genes in the genome is designated as the major (J) strand while the other one as the minor (N) strand. Usually, in most arthropods, the major strand codes for nine PCGs (ND2, ND3, ND6, COI, COII, COIII, ATP6, ATP8, CytB) while the minor strand codes for the other 4 (ND1, ND4, ND4L, ND5). However, the genes that are coded by the two strands in *O*. *oryzae* are different due to the significant movement of both PCGs and the tRNAs in the genome (S4 Table). The minor strand in the *O*. *oryzae* mitogenome codes for ND2, COI, ND6 and CytB, which are almost always present on the major strand in other insect mitogenomes.

### Nucleotide composition

Nucleotide composition of the mitogenome was heavily biased towards A and T with a total A+T content of 85.7% (A = 47.4%, T = 38.3%, C = 8.1% and G = 6.2%) (Table 2). The average A+T content of all the PCGs, tRNAs, rRNAs and the control region were 83.3, 92.2, 88.6 and 93.8%, respectively. Of all the PCGs, the lowest A+T content (76.4%) was found in COI while highest (94.5%) in ATP8. All PCGs on the J (major) strand had a negative AT and GC skew. However, in case of N (minor) strand all the genes had a positive AT skew and a negative GC skew except in case of ATP6 and ATP8 which had positive values for both AT and GC skew (Table 2; S1 Fig). Overall the mitogenome of *O*. *oryzae* had a positive AT skew and a negative GC skew.

**Table 2:** Nucleotide composition of the different genes in the *O*. *oryzae* mitogenome.

Feature	Length	A%	C%	G%	T%	A+T%	Start	Stop	AT Skew	GC Skew
**Whole genome**	15286	47.4	8.1	6.2	38.3	85.7	-	-	0.12	-0.13
**J-Strand**	4093	37.1	9.3	8.5	45.1	82.2	-	-	-0.10	-0.50
**N-Strand**	6677	53.1	9.2	6.8	30.9	84.0	-	-	-0.26	0.15
**PCGs**	10727	43.2	8.7	8.0	40.1	83.3	-	-	0.04	-0.04
**tRNAs**	1552	50.3	4.8	3.0	41.9	92.2	-	-	0.09	-0.23
**N-Strand tRNAs**	585	48.5	3.8	3.6	44.1	92.6	-	-	0.05	-0.02
**J-Strand tRNAs**	967	51.3	5.5	2.7	40.5	91.8	-	-	-0.12	0.34
**ATP6**	678	54.0	6.2	7.8	32.0	86.0	ATG	TAA	0.26	0.12
**ATP8**	147	51.0	3.4	2.0	43.5	94.5	ATA	TAA	0.08	0.09
**COI**	1539	35.7	11.9	11.8	40.7	76.4	ATT	TAA	-0.07	-0.05
**COII**	669	48.8	9.9	9.4	32.7	81.5	ATT	TAA	0.19	-0.02
**COIII**	780	53.1	10.6	8.7	27.6	80.7	ATT	TAA	0.32	-0.1
**CytB**	1128	36.0	9.6	9.5	44.9	80.9	ATT	TAA	-0.11	-0.004
**ND1**	903	53.5	9.6	6.7	30.3	83.8	ATA	TAA	0.28	-0.18
**ND2**	969	39.5	6.8	3.8	49.8	89.3	ATT	TAA	-0.12	-0.28
**ND3**	342	56.7	6.7	5.8	30.7	87.4	ATT	TAA	0.31	-0.05
**ND4**	1185	51.6	10.5	6.4	31.4	83.0	ATA	TAA	0.25	-0.24
**ND4L**	300	54.4	8.0	3.0	35.0	89.4	ATT	TAA	0.22	-0.45
**ND5**	1698	54.7	9.5	5.7	30.2	84.9	ATA	TAA	0.28	-0.25
**ND6**	456	40.6	4.8	4.2	50.4	91.0	ATA	TAG	-0.11	-0.09
**16S rRNA**	1262	45.9	7.4	4.4	42.2	88.1	-	-	-0.005	-0.3
**12S rRNA**	726	44.5	6.9	3.7	44.9	89.4	-	-	0.04	-0.38
**rRNA genes**	1988	45.4	7.2	4.2	43.2	88.6	-	-	0.02	-0.27
**Control Region**	588	40.3	2.4	3.8	53.5	93.8	-	-	-0.14	0.16

### Codon usage and amino acids abundance

**Fig 2:**
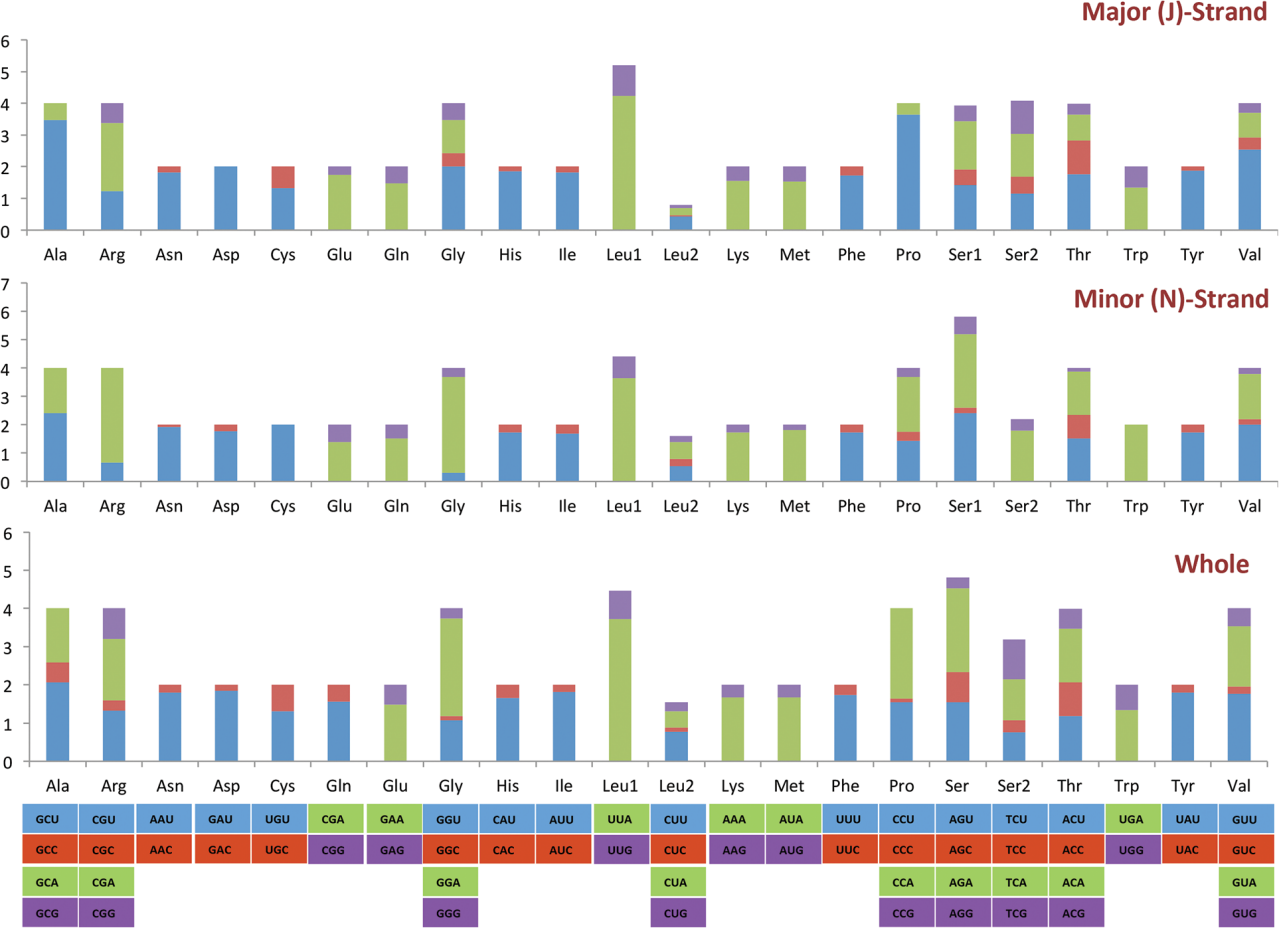
The relative synonymous codon usage (RSCU) values of the J-strand, N-strand and the complete mitochondrial genome of *Orseolia oryzae*. The RSCU values are shown on the Y-axis and the codon families are provided on the X-axis.

The codon usage in the *O*. *oryzae* mitogenome was analysed using the standard invertebrate mitochondrial genetic code and the results are presented in Fig 2. A total of 3573 codons were detected out of which 2209 (61.8%) were found on the J-strand and 1364 (38.2%) on the N-strand. Overall, Phe (TTT,14.6%), Ile (ATT,11.1%), Leu (TTA,8.5%) and Tyr (TAT,6.9%) were the four most commonly represented codons wholly composed of A and/or T, which contributed towards the high A+T content of the entire mitogenome. Together these codons comprised 41.3% of the total codons in the mitogenome. The four-fold degenerate codons showed a bias towards A or T at the third codon position with a preference for A except for isoleucine, valine and alanine. A similar pattern was observed for two-fold degenerate codons at the third codon position, with higher preference for an A/T than G/C indicating an influence of a biased codon usage.

### tRNAs

Considering that this is the first time the *O*. *oryzae* mitogenome sequence is being made available, we considered it pertinent to provide the complete annotation of the mitogenome and therefore, the tRNAs have also been annotated. All the 22 tRNAs, typically found in the arthropod mitochondrial genome, were identified in the *O*. *oryzae* mitogenome, based on primary sequence and secondary structure analyses. However, 6 tRNAs (trn*W*, trn*V*, trn*Q*, trn*Y*, trn*N* and trn*A*) were found to be severely truncated. The D-arm was missing in trn*V* and trn*N* and the T-arm was lacking in trn*W* and trn*A* and was replaced by a TV loop instead of a variable arm and the T-arm. trn*Q* and trn*Y* had a much smaller T-arm and a variable arm, respectively (S2 Fig and S2 File). The variable arm was altogether absent in trn*T*. The predicted secondary structure for trn*H*, trn*L2* and trn*G* revealed much longer variable arms imparting an almost ‘star-like-shape’ to these tRNAs with five radiating arms. However, not all tRNAs showed a truncated structure. Besides, some of the tRNAs (between trn*Q* and trn*M*, between all tRNAs in the region between COI and ND3 (except for trn*Y*) and between trn*T* and trn*P*) were found to have overlapping sequences. Though specific reasons for the truncation in mitochondrial tRNAs are unknown, it has been hypothesized that these may be due to the evolutionary pressure for mitochondrial genomes to attain smaller size [22].

The present study indicated the presence of two trn*L*, located between trn*I* and COI, which are probably non-functional due to the occurrence of introns in the anti-codon loop region. Given that a functional copy of the tRNA is also present at its canonical position, it is possible that the two above-referred trn*L*s may be a molecular relic of rearrangement event(s) that may have taken place in the evolutionary history of the rice gall midge. Similar non-functional copies have been previously reported in other insect mitogenomes. An additional copy of trn*L* (uag) was found between trn*S* (uag) and ND1 in the *Cryptopygus antarticus* mitochondrial genome [23] where this copy was reported to have several mismatches in the acceptor arm hindering proper folding of the tRNA. Further, the authors speculated that this could be the trace of a duplication event that occurred earlier during the course of its evolution.

### Ribosomal RNAs

The ends of the rRNA genes in *O*. *oryzae* were assumed to extend to the boundaries of the flanking genes on both sides, as it was not possible to accurately determine them by sequencing. The 16S rRNA (large rRNA subunit) was found to be present between trn*L1* and trn*V* while the 12S rRNA (small rRNA subunit) was present between trn*V* and the control region. The location of the rRNA genes in the *O*. *oryzae* mitogenome was similar to that observed in the ancestral gene order in arthropods. The 16S rRNA was 1,262 bp long with an A+T content of 88.1%, while 12S was 726 bp with an A+T content of 89.4%. Both the rRNA genes put together had a positive AT skew and a negative GC skew (Table 2). Unlike other arthropod genomes, where rRNA genes are coded on the minor strand, the rRNA genes in the *O*. *oryzae* mitogenome were coded on the major strand, a trait previously reported only in *Thrips imaginis* [24].

### Repeat Regions

**Fig 3:**
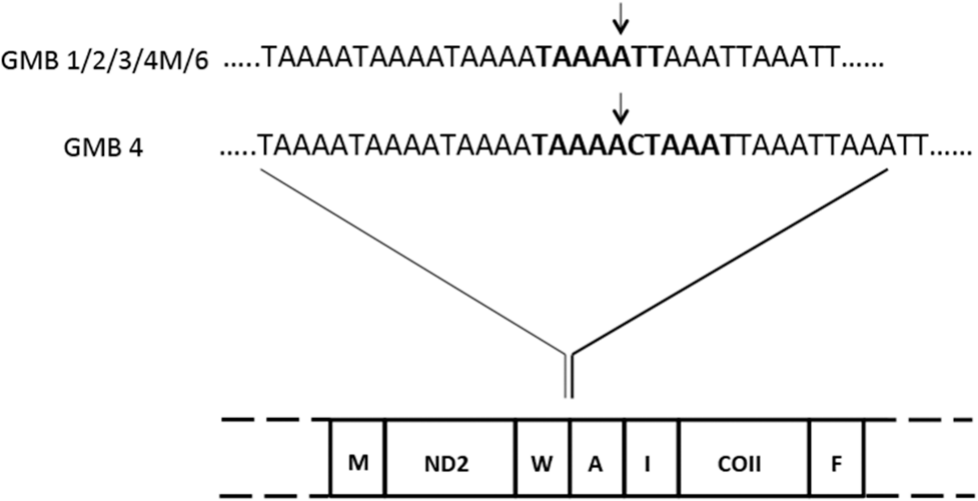
Location of the repeat region I (RR-I) in the *Orseolia oryzae* mitogenome. Arrows indicate change in repeat motifs in RR-I in different biotypes of *O*. *oryzae*.

Tandem repeats were detected in the *O*. *oryzae* mitogenome at two different non-coding regions. One was observed between trn*W* and trn*A* (Repeat Region I) and the other between trn*I* and COI (Repeat Region II) (Figs 1 and 3)–a feature not reported in any other arthropod mitogenomes sequenced thus far.

### Repeat region I (RR-I)

This region (RR-I) was located in the intergenic spacer between trn*W* and trn*A*. In this non-coding region we observed two different penta-nucleotide tandem repeats with motifs ‘TAAAA’ and ‘AAATT’ in the case of GMB1, GMB2, GMB3, GMB4M and GMB6. Though GMB4 showed presence of two penta-nucleotide repeats, the second repeat motif changed from ‘AAATT’, as found in other biotypes, to ‘TAAAT’ due to a transition of T to C (Fig 3). Of the 7 biotypes analysed, 6 (GMB1, GMB2, GMB3, GMB4, GMB4M and GMB6) harboured this repeat region while this region was absent in GMB5 (Table 3). RR-I was also absent in the African gall midge and the *Paspalum* midge. However, in GMB3, even though both repeat motifs were present, these motifs were interrupted by non-repetitive sequences unlike in other biotypes. In addition, it was observed that the iterations of these repeats elements differed among all rice gall midge biotypes studied (Table 3).

**Table 3:** Number of iterations of repeat motifs in RR-I across *O*. *oryzae* biotypes and also in different species of *Orseolia*.

Biotype/Species	Repeat Motif		
	TAAAA	AAATT	TAAAT
**GMB 1**	25	49	-
**GMB 2**	11	61	-
**GMB 3**	11+5*	16+29*	-
**GMB 4**	44	-	30–33
**GMB 4M**	41	28–37	-
**GMB 5**	-	-	-
**GMB 6**	5–7	52–58	-
***Orseolia oryzivora***	-	-	-
***Orseolia fluvialis***	-	-	-

(-: repeat motif absent

*: interrupted repeat)

### Repeat region II (RR-II)

The second repeat region (RR-II) in the gall midge mitogenome was located between trn*I* and COI. The repeat motif identified was ‘ATTTATATTTAA’ and was detected in 4 of the 7 biotypes studied. This motif was present as a perfect repeat in all biotypes except in GMB6 where the motif changed to ‘ATTTATATCTAA’ in two iterations due to a transition of T to C. RR-II was altogether absent in GMB2, GMB3, GMB5, African gall midge and the *Paspalum* midge (Table 4).

**Table 4:** Number of iterations of repeat motifs in RR-II across *O*. *oryzae* biotypes and also in different species of *Orseolia*.

Biotype/Species	No. of Repeats
**GMB 1**	5.4
**GMB 2**	None
**GMB 3**	None
**GMB 4**	3.4
**GMB 4M**	4.4
**GMB 5**	None
**GMB 6**	5.4*
***Orseolia oryzivora***	None
***Orseolia fluvialis***	None

(*Interrupted repeat)

Interestingly, these unique tandem repeats, previously unreported in any of insect mitogenomes sequenced thus far, were found in the segment of the gall midge mitogenome that showed maximum rearrangements. These repeats could possibly be the remnants of some transpositional activity leading to shuffling of genes in the mitogenome. Replication slippage and intra-mitochondrial recombination could also explain the presence of these tandem repeats [25,26].

Mitochondrial DNA sequences have been used in the past to identify interspecies and intraspecific nucleotide polymorphism and gene length differences. These studies have predominantly relied on COI, CytB, 16S and 12S as molecular markers. However, various reports reveal that species identification based on 16S and 12S can yield different results [27] as phylogenetic trees inferred using these two sequences can generate evolutionary relationships that are not similar. Hence, species identification and nucleotide polymorphism studies based on 16S or 12S sequences are likely to be inaccurate as well as inconclusive. Moreover, above-mentioned markers are not useful in biotype differentiation.

Currently, the method for biotype differentiation is the use of genetic differentials using rice varieties that harbour different R genes for gall midge resistance [3]. The type of reaction observed (susceptibility or resistance) between the specific plant genotype and the insect genotype determine the biotype status of the insect. This method of biotyping is tedious and time consuming (21–35 days).

Tandem repeats are frequently observed in plant nuclear and chloroplast genomes. In the past, various tandem repeats have been discovered in plants that have been successfully exploited to detect population genetic structure [28,29,30]. However, to the best of our knowledge, repeats in the mitogenome have never been exploited for biotype differentiation in insects. However, with the discovery and subsequent exploitation of this feature, it would help expedite biotype differentiation in a short period of time using PCR and without the need for the host. More importantly, these assays could be performed on single insects without need for an insect population a prerequisite when biotyping using the earlier referred, laborious and time-consuming differential screening method.

Our results show that the repeat region was present in 6 of the 7 Indian biotypes studied but was absent in GMB5, African rice gall midge (*O*. *oryzivora*) and the *Paspalum* midge (*O*. *fluvialis*). Therefore, clearly indicating that these repeats provide a valuable tool not only to distinguish the different biotypes of the gall midge but also to differentiate *Orseolia* species. In addition, during the course of this study, we observed heteroplasmy (with reference to the number of iterations in the repeat region RR-I) within individuals of biotypes (data not shown). Heteroplasmy has been previously recorded with reference to repeat sequences in the control region of the mitochondrial genomes of insects e.g. bark weevils [31]. However, in the present study, heteroplasmy was observed in relation to one of the two repeat regions found in the intergenic spacers of the mitochondrial genome of *O*. *oryzae*.

As the same pair of primers was able to amplify the regions harbouring RR-I and RR-II across all biotypes and two species of *Orseolia*, it indicates that there is enough sequence conservation in the regions flanking these repeat regions for these primers to bind. Hence, these primers can be regarded as ‘Universal Primers’ for the PCR amplification of these repeats in the rice gall midge. Moreover, the presence of two different sets (motifs) of penta-nucleotide repeats in this region makes the detection and scoring of these markers more accurate and therefore, making the identification of biotypes and differentiation between *Orseolia* species, more precise. Besides providing a quick and dependable method for the identification of biotypes, it will aid in developing a reliable early warning system with regard to prevalence of a particular biotype in a rice growing area. And in addition, our ability to quickly and correctly identify biotypes will have relevant implications in the area of integrated management of pests [32].

In spite of the fact that differences in repeat regions could distinguish all the biotypes tested, we do not suggest any linkage between the marker phenotype and the virulence gene in the insect. It is likely that repeat region polymorphism is related to geographical distribution of the insect populations that represented the biotypes used in this study. Also, some of the virulence genes in the insect are sex-linked and materially inherited [33,34] and therefore, these mitogenome markers could also be detecting the virulence trait indirectly. This however, needs further investigation. Earlier, attempts have been made to distinguish gall midge biotypes through Random Amplified Polymorphic DNA (RAPD) [15] or Simple Sequence Repeat (SSR) [35] markers. But these markers were not very consistent when tested on a large and varied population. More studies involving inheritance of these mitochondrial markers and co-segregation with virulence phenotype in mapping populations are needed to improve the reliability of these markers in biotype differentiation.

### Control Region

**Fig 4:**
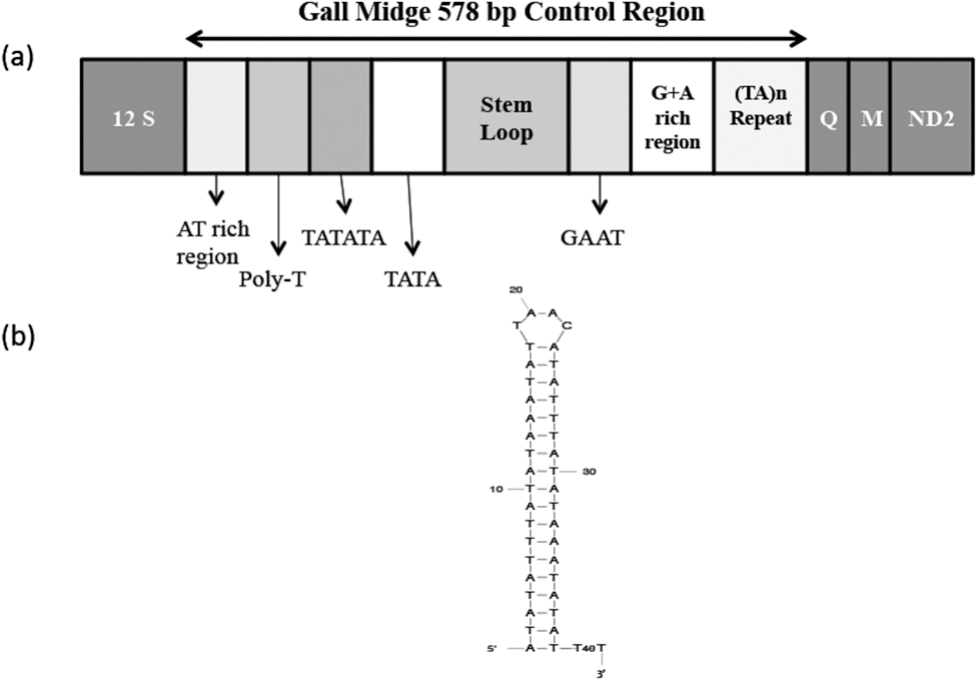
The control region of *Orseolia oryzae* mitochondrial genome. (a) The structural elements in the control region. The regions flanking the control region i.e. 12S and *trnQ*, *trnM* and ND2 are represented by the dark grey boxes (b) The secondary structure of the putative stem loop found in the control region.

The control region for *O*. *oryzae* was found to be 578 bp long with an A+T content of 93.8%. Analysis of the region revealed five structural elements conserved in other arthropods as well ie: a poly-T, a [TA(a)]n-like-structure, a highly conserved stem loop structure, a pair of sequences immediately flanking the stem loop with a 5’ consensus of ‘TATA’ and 3’ consensus of ‘G(A)nT’ and finally a G+A rich region downstream of the secondary structure (Fig 4). A comparison of these structural elements was carried out with other members of Diptera (S5 Table). All the above five structural elements of the control region were found to be present in the conserved order in the *O*. *oryzae* mitogenome, as observed in other arthropod genomes, and on the same strand. Though the five conserved elements are present in all the dipteran insects analysed, the 3’ consensus motif, G(A)nT, was lacking in *C*. *quinquefasciatus*.

Furthermore, an inverted repeat of 144 bp was also found in the control region of *O*. *oryzae*. A poly-T was present 5’ to the secondary structure in all the insects compared except in *D*. *yakuba* where its location was 3’. The poly-T is thought to be associated with the replication origin or with the control of transcription. Another interesting feature is the presence of a highly AT rich domain with A+T content ranging between 95–99% across the different insects compared (S6 Table).

The control region of the different biotypes was checked for the presence of repeats. Two types of repeats were found in the control region of GMB1, GMB4, GMB4M and GMB6. One a TA repeat and the other a longer 97 bp repeat in GMB1, GMB4M and GMB6; and 103 bp long repeat in GMB4 (S7 Table). Therefore, the respective lengths of the control region in all the four biotypes were also different. However, the control region from GMB5, *O*. *oryzivora* and *O*. *fluvialis* could not be amplified and therefore, not studied.

The control regions of the four different biotypes were also analysed for the presence of the five conserved structural elements known to occur in the arthropod control region. The four biotypes studied showed the presence of all five structural features in their respective control regions (S8 Table). While the A+T content of the control regions was near identical in the four biotypes, the 5’ and 3’ consensus sequences were identical. When the nucleotide sequence of the control region was compared amongst the four biotypes, it was observed that all the stem loops were 42 bp long and were almost identical (>95%) in terms of nucleotide sequence (S3 File). The only major difference in the control region between the four biotypes was in the number of the ‘TA’ repeats.

### Reversal of strand asymmetry

Usually for most of the insect mitogenomes, the major strand has positive AT skew values and negative GC skew values implying that there is strand asymmetry with an excess of A compared to T and an excess of C compared to G on the major strand. However, it has been found that reversal in strand asymmetry, in mitogenomes, is indicative of mitogenomes that have undergone rearrangement [36] as observed in the rice gall midge mitogenome as well.

An earlier study [36] suggested that reversal of strand asymmetry is probably due to the inversion of the control region. Strand asymmetry is assumed to be the result of deamination of A and C bases in single-stranded DNA during the process of replication and transcription. However, the level of contribution of each process remains debatable. If it is assumed that replication plays the major role, then inversion of the origin of replication, in the control region, would lead to the reversal of strand asymmetry. Wei et al. [36] determined the direction of the elements involved in the regulation of replication and transcription in 10 different species belonging to families Braconidae, Aleyrodidae and Philopteridae that showed reversal of strand asymmetry. Further, the analysis revealed that the elements were indeed present in different directions and strands; hence, leading to the reversal of strand asymmetry. A similar analysis was carried out for the *O*. *oryzae* control region. All these elements were found to be present in the conserved direction and strand. Therefore, indicating that a mechanism other than inversion of the origin of replication could be responsible for reversal of strand asymmetry in the rice gall midge mitogenome.

### Rearrangements

**Fig 5:**
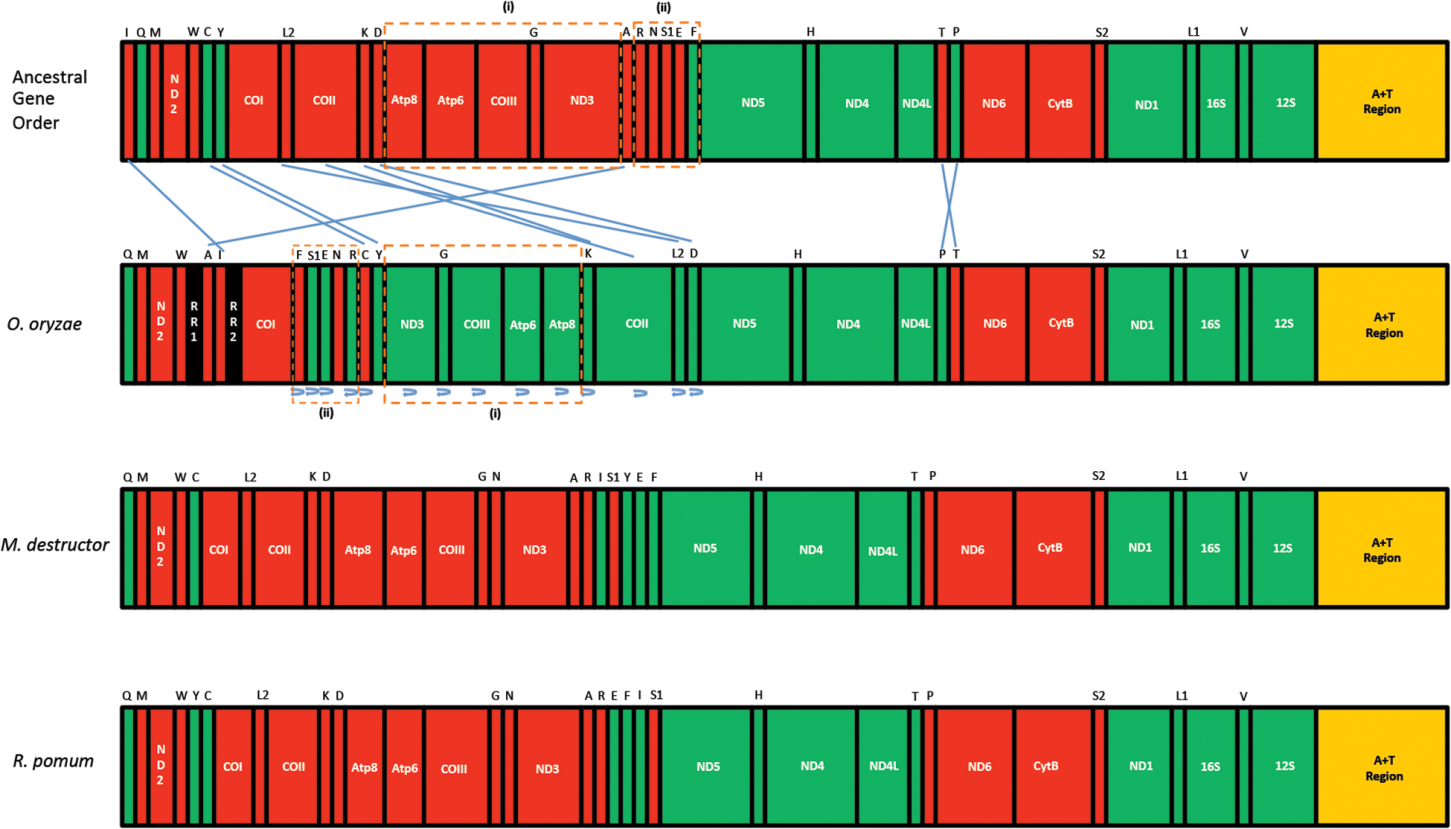
Gene rearrangements in *O*. *oryzae* mitogenome. The order of genes in the *Orseolia oryzae* mitochondrial genome relative to the ancestral order and two other gall midges, *Mayetiola destructor* [GQ387648] and *Rhopalomyia pomum* [GQ387649].

In the *O*. *oryzae* mitogenome, all the three major classes of gene rearrangements were observed i.e. translocation, local inversion (inverted in the local position) and shuffling and remote inversion (translocated and inverted) (Fig 5). Rearrangements involved a total of 5 PCGs and 15 tRNAs which were either translocated and/or inverted. Relative to the conserved gene order, only 14 ancestral gene boundaries were conserved in the genome. Furthermore, it was observed that maximum rearrangements were restricted to one portion of the genome i.e. the part of the genome upstream of ND5. Downstream of ND5 only one rearrangement was observed: the translocation of trn*T* and trn*P* but without inversion. Among all the rearrangements, two gene blocks could be identified that were translocated and inverted *en masse* with reference to the ancestral gene order. One such rearrangement involved both PCGs and tRNAs; ATP8-ATP6-COIII-trn*G*-ND3 to ND3-trn*G*-COIII-ATP6-ATP8 and the other comprising only tRNAs; trn*R*-trn*N*-trn*S1*-trn*E*-trn*F* to trn*F*-trn*S1*-trn*E*-trn*N*-trn*R* (Fig 5).

In contrast to plants, animal mitogenomes are very compact and the proportion of coding versus non-coding sequences is high. The number and size of the intergenic spacers are usually limited and their presence is linked to errors during replication of the mitogenome i.e. duplication of portions of the genome, with supernumerary copies becoming pseudogenes and gradually disappearing. Another distinguishing feature in the genome is the lengths of the intergenic spacers. Usually, these spacers are not longer than 100 bp except in the case of the control region. This agrees with the overall economy of size of the mitogenome. Owing to this, the movement of genes would be a rare event as genes would have to be ‘cut and pasted’ with high accuracy. If at all these cuts were to happen at places other than the gene borders it could prove to be lethal [37]. Hence, more the number of rearrangements more would be the number of lethal events. However, in the *O*. *oryzae* mitogenome intergenic spacers as long as 397 bp are present. Also, the percentage of these regions, relative to the genome size, is very high as compared to other dipterans (S3 Fig) where no such rearrangements have taken place compared to the ancestral order. These relatively longer intergenic spacers probably help in accurate ‘cutting and pasting’ of the genes facilitating movement of genes from their ancestral positions. Consistent with this is the fact that gene order in dipteran mitogenomes, with lower percentage of intergenic spacers, has remained highly stable over long evolutionary periods (S4 Fig). Nevertheless, it has also been observed that in some lineages, with low percentage of intergenic spacers in their mitogenomes, the rate of gene rearrangement is very high, e.g. Hymenoptera (bees, wasps and ants) [38,39,40] and the hemipteroids (lice and thrips) [41,42], and exhibit a highly divergent order compared with the conserved arthropod organization. However, a few hymenopterans and hemipteroids do possess the ancestral insect mitogenome gene order [43]. Similarly, *O*. *oryzae* is the only dipteran, sequenced thus far, to have such a highly rearranged mitogenome. Previous studies indicate that the rearrangements in this order are restricted only to tRNAs eg. inversion of trn*S1* in the family Culicidae [12,13] and the inversion of trn*T* and trn*P* without any translocation as observed in other gall midges from the family Cecidomyiidae [38]. In addition, *O*. *oryzae* shows an inversion of trn*S1* and a translocation of trn*T* and trn*P* but without any inversion (Fig 5).

Most of the rearrangements in mitogenomes have been explained by the tandem duplication-random loss (TDRL) mechanism. Two possible models (predicted using the CREx software) could be used to elucidate the different steps that could have lead to the present gene order in the *O*. *oryzae* mitogenome from the ancestral arthropod gene order (S5 Fig). However, it is slightly hard to conceptualize these models based on duplication and random loss. First of all, duplications would lead to much larger mitogenomes, which due to longer replication times would be of a selective disadvantage to the organism. Secondly, random loss of DNA portions that have been duplicated would take substantial time as the surplus genes would be removed through microdeletions that are introduced only at the time of replication [37]. However, the above factors would not hold true if the rearrangements were explained on the basis of intramitochondrial recombination, as through this model the deletion events would occur relatively fast. Also, recombination can better explain the inversion of so many genes in the *O*. *oryzae* mitogenome that has lead to reversal of strand asymmetry. In an earlier study involving human mitochondria, Kajander et al. [26] found the presence of mitochondrial DNA molecules with rearrangements present at low levels, in healthy human subjects. The authors designate these molecules as ‘sublimons’. However, the proportion of mitochondria with rearrangements, as compared to ancestral order, was found to be much higher in cells, such as those of the heart or skeletal muscle tissue, that are frequently exposed to oxidative stress. The gall midge spends a considerable period of its life feeding inside the host rice plant. During the larval stages, the feeding maggots are likely to be constantly exposed to the host defence molecules that create an environment of oxidative stress for the larvae. It is probably at this point that the mitochondrial DNA is more prone to rearrangements which in turn probably provide the insect a selective advantage in its battle to overcome the hostile environment within the host.

Initially, it was thought that rearrangements in the mitogenome and the parasitic mode of life are linked. This hypothesis was later rejected as only Hymenoptera and the hemipteroid orders showed rearranged mitochondrial sequences, while the mitogenomes of parasitic and non-parasitic Diptera showed no rearrangements [44,45]. However, this hypothesis cannot be completely rejected as *O*. *oryzae* is a parasitic arthropod, belonging to the order Diptera, with a rearranged mitochondrial genome just as in the case of the three hemipteroid orders with rearranged mitochondrial genomes. As discussed earlier, the rice gall midge is likely to encounter oxidative stress while feeding on the host plant. Similarly, the other parasitic arthropods (Hymenoptera and hemipteroid orders) are also likely to be exposed to a similar stress leading to rearrangements in the gene order through intramitochondrial recombination. It is probable, therefore, that the parasitic mode of life led to highly rearranged mitogenomes in these insect orders.

### Phylogenetic Relationships

**Fig 6:**
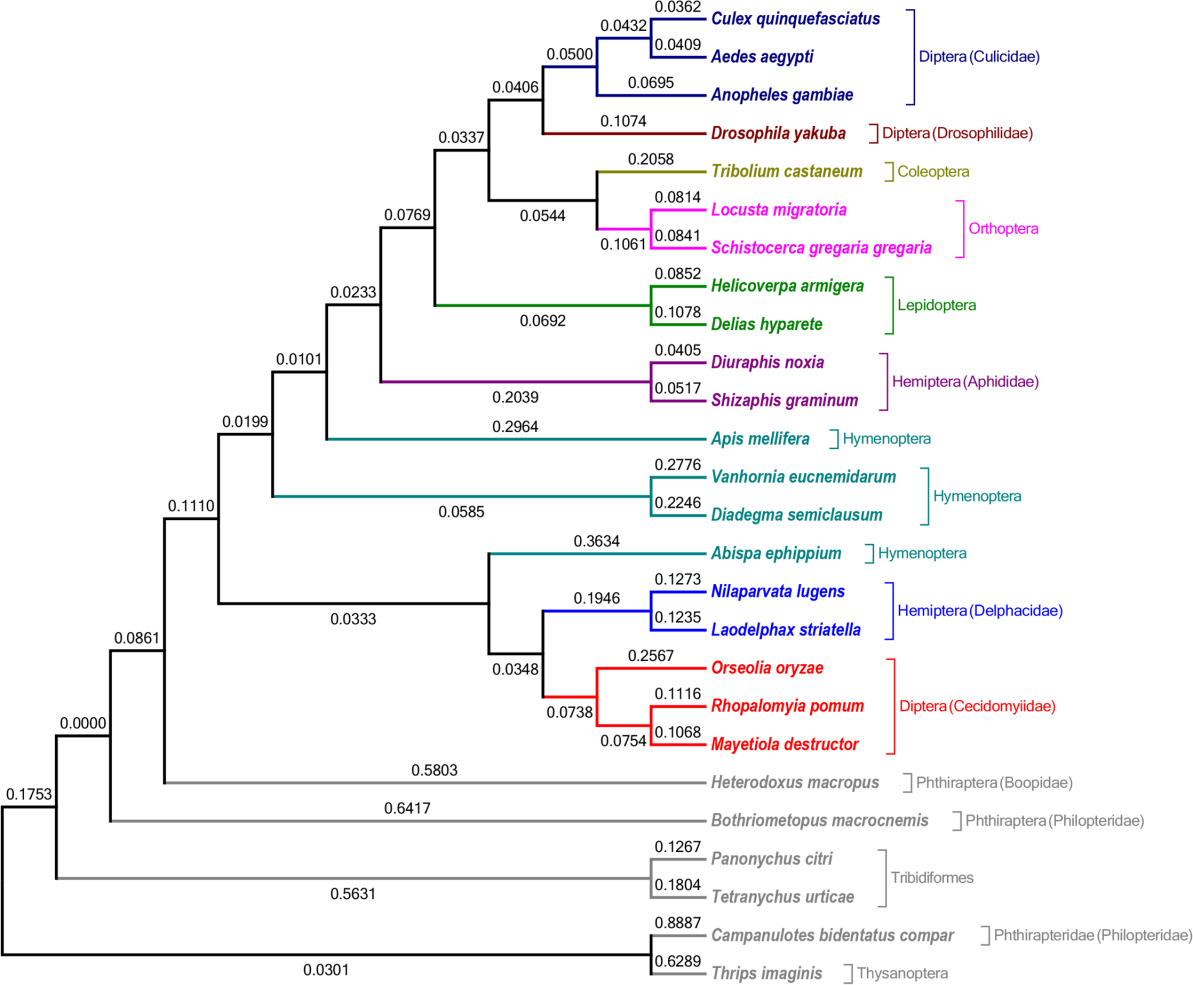
Phylogenetic tree inferred from the multiple sequence alignment of the complete mitogenomes of 26 arthropods (see Materials and Methods for details). The tree was constructed using the Maximum Likelihood method and pair-wise distances were calculated using the Tamura-Nei method included in MEGA. The numbers at nodes indicate genetic change. The tree was arrived at using 1000 bootstrap replications.

Phylogenetic analysis was performed with a large data set comprising 26 arthropod species for which complete mitogenome sequences are available (Fig 6). The complete mitochondrial genome sequence of each species was used to construct the consensus phylogenetic tree using the Maximum Likelihood method. The entire sequence of the genome (including the 13 PCGs, 22 tRNAs, 2 rRNAs and the control region) was used as it gives a better understanding of phylogeny with improved nodal confidence [46]. The analysis included insects from various orders: Diptera, Lepidoptera, Coleoptera, Orthoptera, Hemiptera, Hymenoptera, Phthiraptera and Thysanoptera. Seven dipteran members were involved in the analysis including 3 species each of mosquito (Culicidae) and gall midge (Cecidomyiidae) and *D*. *yakuba* (Drosophilidae) was used as the seventh member from this order. In the phylogenetic tree, Diptera formed two separate clusters. One cluster constituted members of the family Culicidae (mosquitoes; haematophagous) and the other members of the family Cecidomyiidae (gall midges; phytophagous). *O*. *oryzae* had a much longer branch length than that of *M*. *destructor* or *R*. *pomum* indicating a much higher evolutionary rate in the rice gall midge mitogenome compared with the other two gall midges. Also, the gall midges formed a clade that was placed much closer to the insect species with highly rearranged mitogenome belonging to the hemipteroid orders. The gall midges formed a subtree with brown plant hoppers (Hemiptera; Delphacidae) that have a similar feeding habit. Interestingly, individuals belonging to Phthiraptera and Thysanoptera formed separate branches in the tree, as these are families with members that exhibited highly rearranged mitochondrial genomes.

## Conclusions

There are four main reasons why the Asian rice gall midge can be used as a model organism for studying evolution of arthropod mitogenomes. First, it has a unique repeat region that has never been observed in any of the insect mitogenomes sequenced so far. Second, the genome exhibits different kinds of rearrangements: inversions and translocations of genes through wide distances. Third, it is the only dipteran that has rearrangements involving PCGs as well as tRNAs and fourth, it shows truncation in the secondary structures of the tRNAs as already reported in the case of other cecidomyiids [14] and most of the parasitic arthropods [47]. Various questions can be answered by studying the *O*. *oryzae* mitogenome i.e. (i) What is the mechanism responsible for rearrangements in the mitogenome? (ii) Do the tandem repeats found in the genome have any role to play in this rearrangement process, and if yes, then what could be the possible mechanism? (iii) What is the evolutionary significance that *O*. *oryzae* is the only parasitic arthropod in the order Diptera to have a rearranged gene order? (iv) Can this rearrangement be used to gather phylogenetic correlation between Diptera and the three hemipteroid orders that show rearrangements?

## Supporting Information

S1 Fig Strand asymmetry of the *Orseolia oryzae* mitochondrial genome. (a) Nucleotide composition of the all PCGs and the rRNAs of the *O*. *oryzae* mitochondrial genome and (b) AT- and GC-skew values of the PCGs and the rRNAs.
(PDF)

S2 Fig The predicted secondary structures of the 22 tRNAs identified in the *Orseolia oryzae* mitochondrial genome. (-) indicate Watson-Crick bonds, (=) indicate bonds between C and G, (●) indicate bonds between similar residues and hollow circles (○) indicate bonds between U and G. The last secondary structure illustrates each stem and loop in the tRNAs: AA-arm for amino acid acceptor arm, T-arm for TΨC arm, V-arm for variable arm, AC-arm for anticodon arm, and D-arm for dihydrouridine arm.
(PDF)

S3 Fig The relative percentage of intergenic spacers, rRNAs, tRNAs and the control region as a function of mitogenome size across different insects in Diptera. (PDF)

S4 Fig Comparison between the control regions of different *Orseolia oryzae* biotypes. (a) The predicted secondary structure of the putative stem loop region in the control region in different biotypes. (b) Multiple sequence alignment of the putative stem loop region sequences in the control region of different biotypes (GMB1, GMB4, GMB4M and GMB6). Asterisks indicate identical nucleotide residues.
(PDF)

S5 Fig Predicted mechanisms of gene order rearrangements in the rice gall midge mitochondrial genome. The two predicted models (I and II) represent two different series of steps inferred using the CREx software. The scenario predicted in Model I require two Reversals, one transposition and one TRDL while Model II shows two Reversals and two TRDLs to arrive at the current mitochondrial gene order found in *Orseolia oryzae* from the ancestral arthropod gene order.
(PDF)

S6 Fig Comparison of the control region of different dipterans illustrating the various structural elements. The regions flanking the control region i.e. 12S and *trnQ*, *trnM* and ND2 are represented by dark grey boxes (a: *Drosophila yakuba*, b: *Rhopalomyia pomum*, c: *Mayetiola destructor*, d: *Culex quinquefasciatus*, e: *Anopheles gambiae*).
(PDF)

S1 File Non-coding regions in the gall midge and comparison of different PCG lengths in dipteran mitogenomes. (PDF)

S2 File Truncated tRNAs. (PDF)

S3 File Control Region. (PDF)

S1 Table List of primers used to amplify the 18 overlapping fragments in the *Orseolia oryzae* mitogenome. (PDF)

S2 Table Accession numbers of all the species used for comparison in the present study. (PDF)

S3 Table A comparison of start and stop codons for PCGs across different species in Diptera. (PDF)

S4 Table Distribution of PCGs and tRNAs on the major (J) and minor (N) strands across different species in Diptera. (PDF)

S5 Table Composition of the control region across Diptera. (PDF)

S6 Table A Comparison of the repeats present in control region of different species of Diptera. (PDF)

S7 Table Number of repeats present in the control region of different biotypes of *Orseolia oryzae*. (PDF)

S8 Table Organization of the control region in different biotypes of *Orseolia oryzae*. (PDF)

S9 Table The location of the mtTERM sequence across different species in Diptera. (PDF)
