# Supplementary figures and images for: The Asian Rice Gall Midge (Orseolia oryzae) Mitogenome Has Evolved Novel Gene Boundaries and Tandem Repeats That Distinguish Its Biotypes
Source: PLoS One. 2015 Jul 30;10(7):e0134625. doi: 10.1371/journal.pone.0134625 (PMC4520695; doi:10.1371/journal.pone.0134625)

(a)

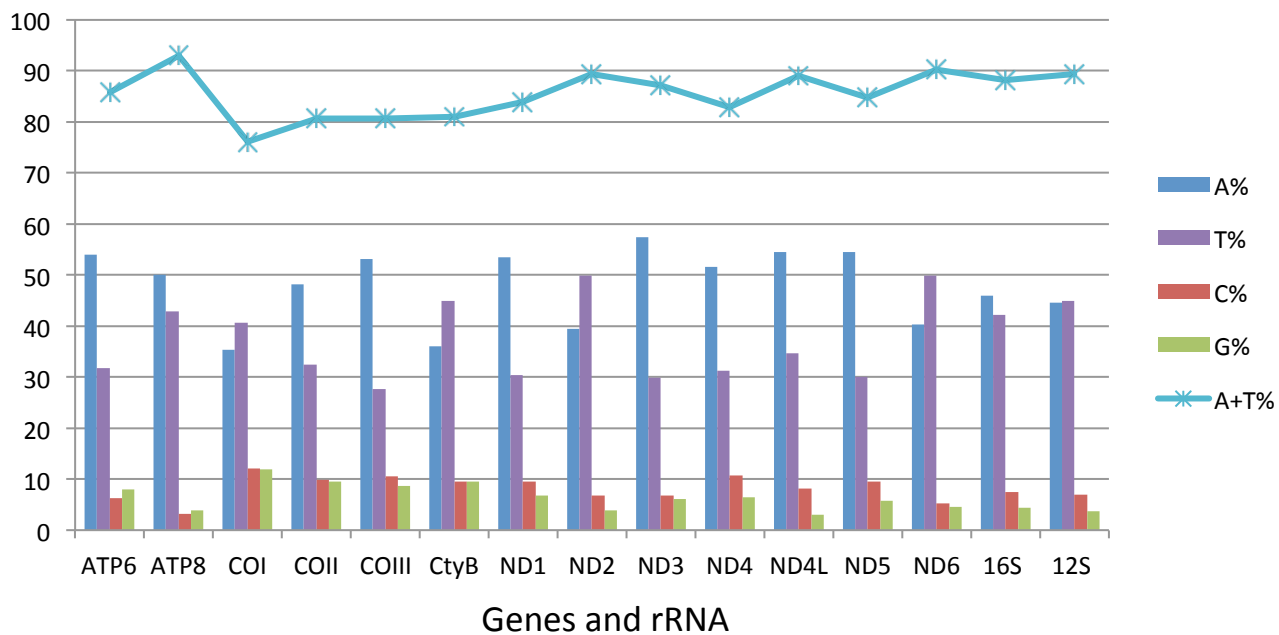

(b)

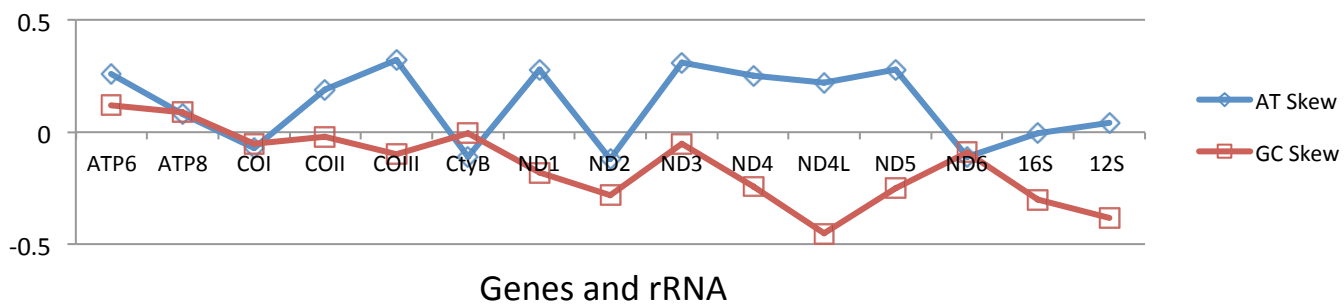

Supplement: S1 Fig — (a) Nucleotide composition of the all PCGs and the rRNAs of the O. oryzae mitochondrial genome and (b) AT- and GC-skew values of the PCGs and the rRNAs. (PDF) [file pone.0134625.s001.pdf]

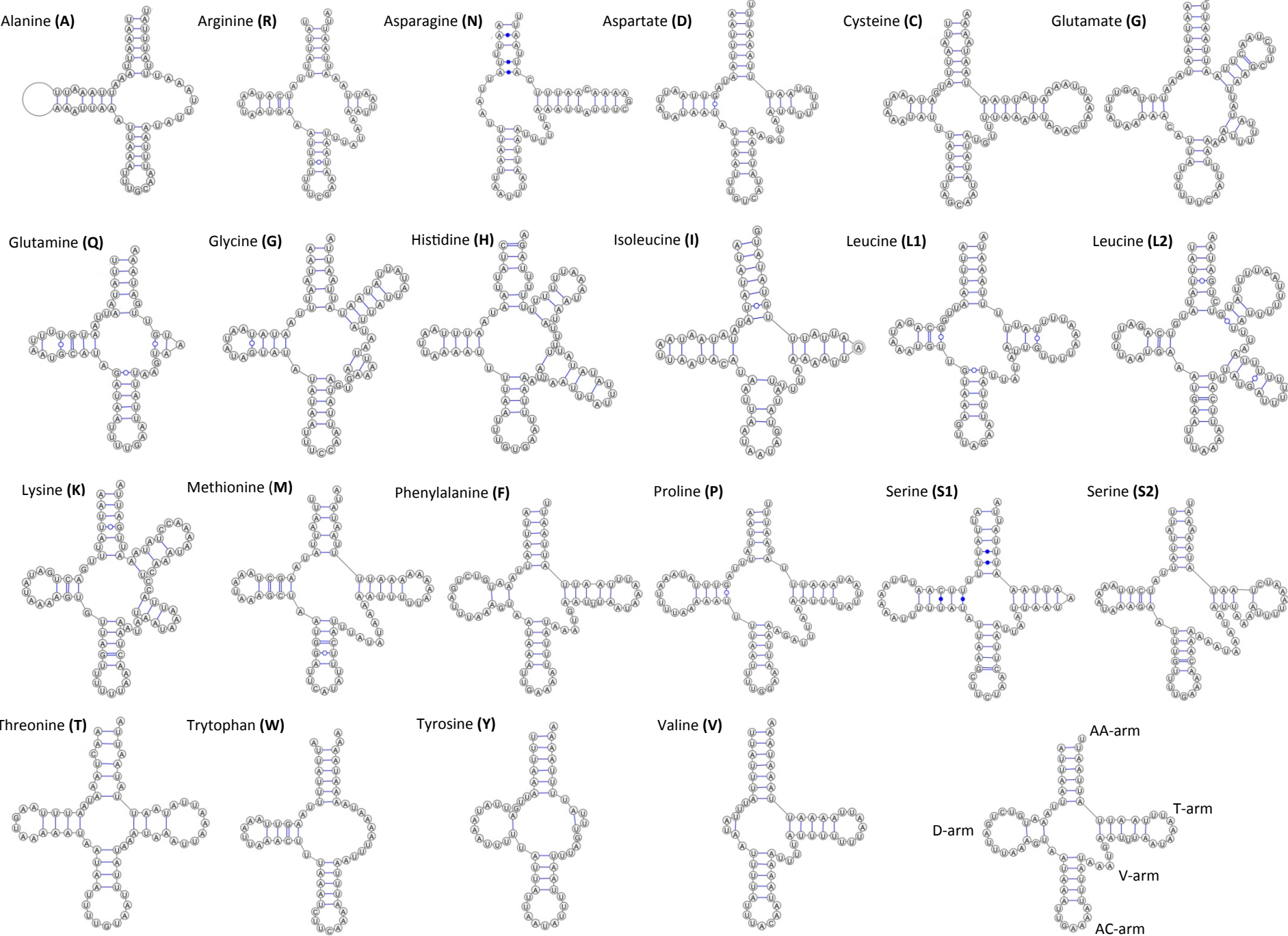

Supplement: S2 Fig — (-) indicate Watson-Crick bonds, (=) indicate bonds between C and G, (●) indicate bonds between similar residues and hollow circles (○) indicate bonds between U and G. The last secondary structure illustrates each stem and loop in the tRNAs: AA-arm for amino acid acceptor arm, T-arm for TΨC arm, V-arm for variable arm, AC-arm for anticodon arm, and D-arm for dihydrouridine arm. (PDF) [file pone.0134625.s002.pdf]

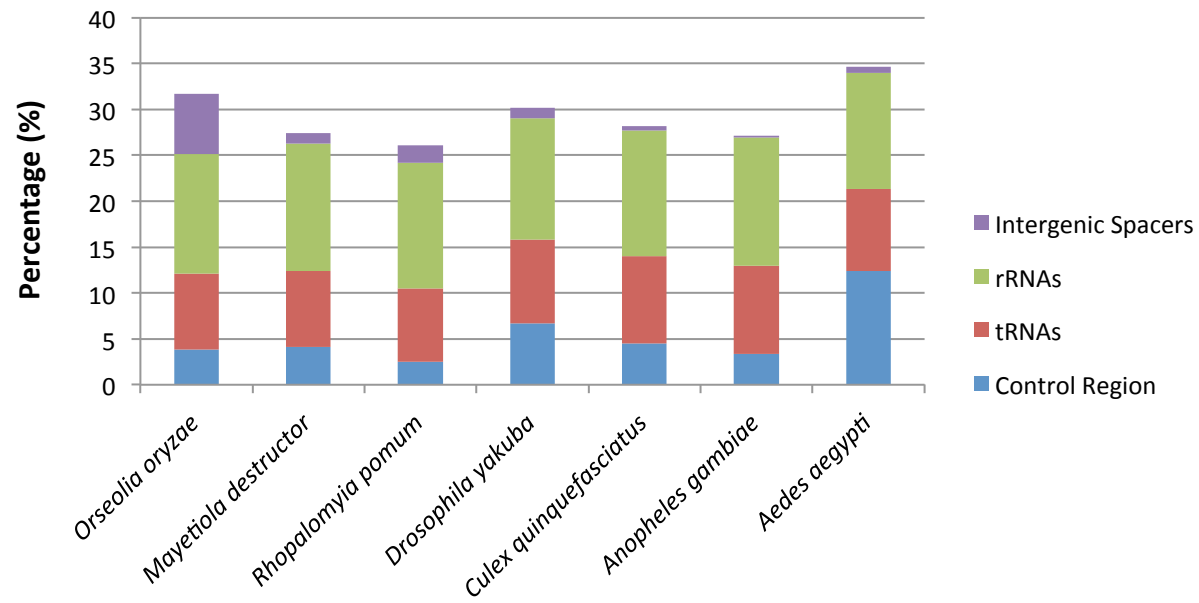

Supplement: S3 Fig — (PDF) [file pone.0134625.s003.pdf]

### Model (I)

## Reversal

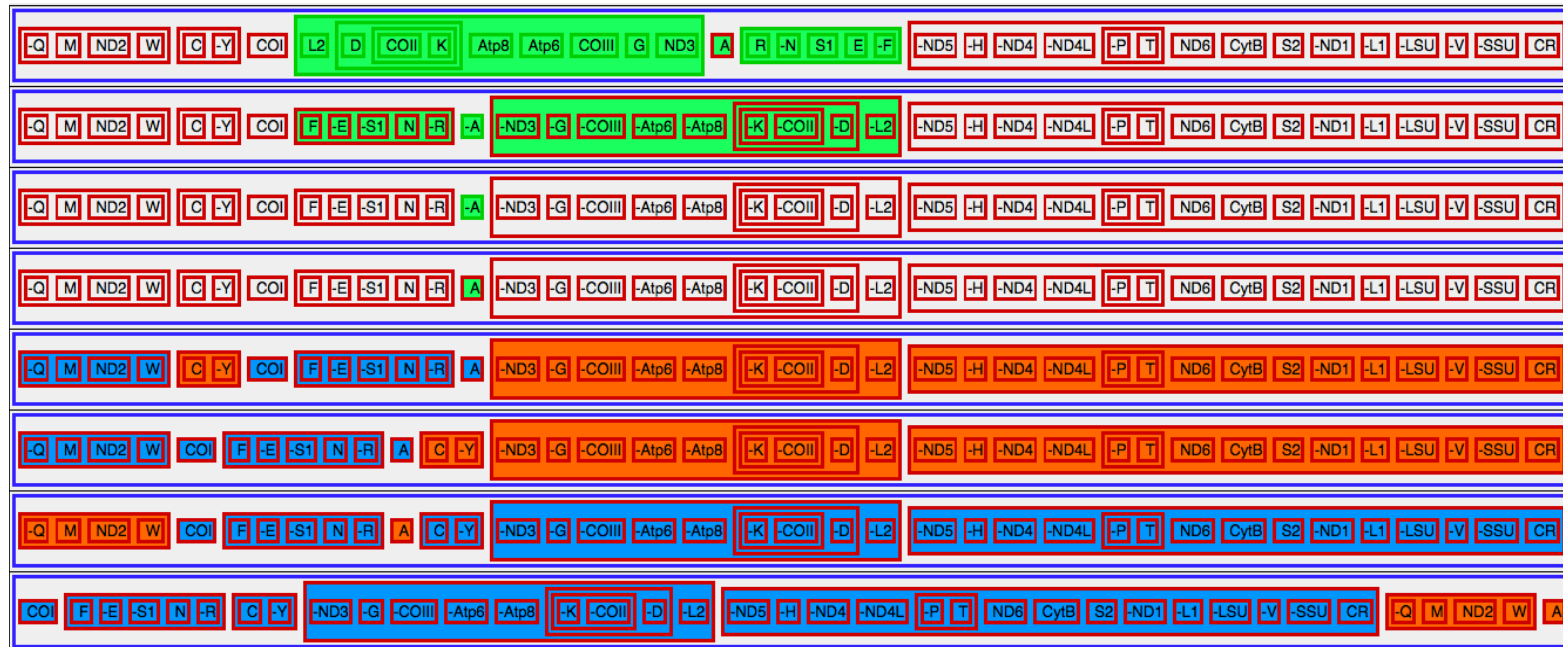

### Model (II)

## Reversal

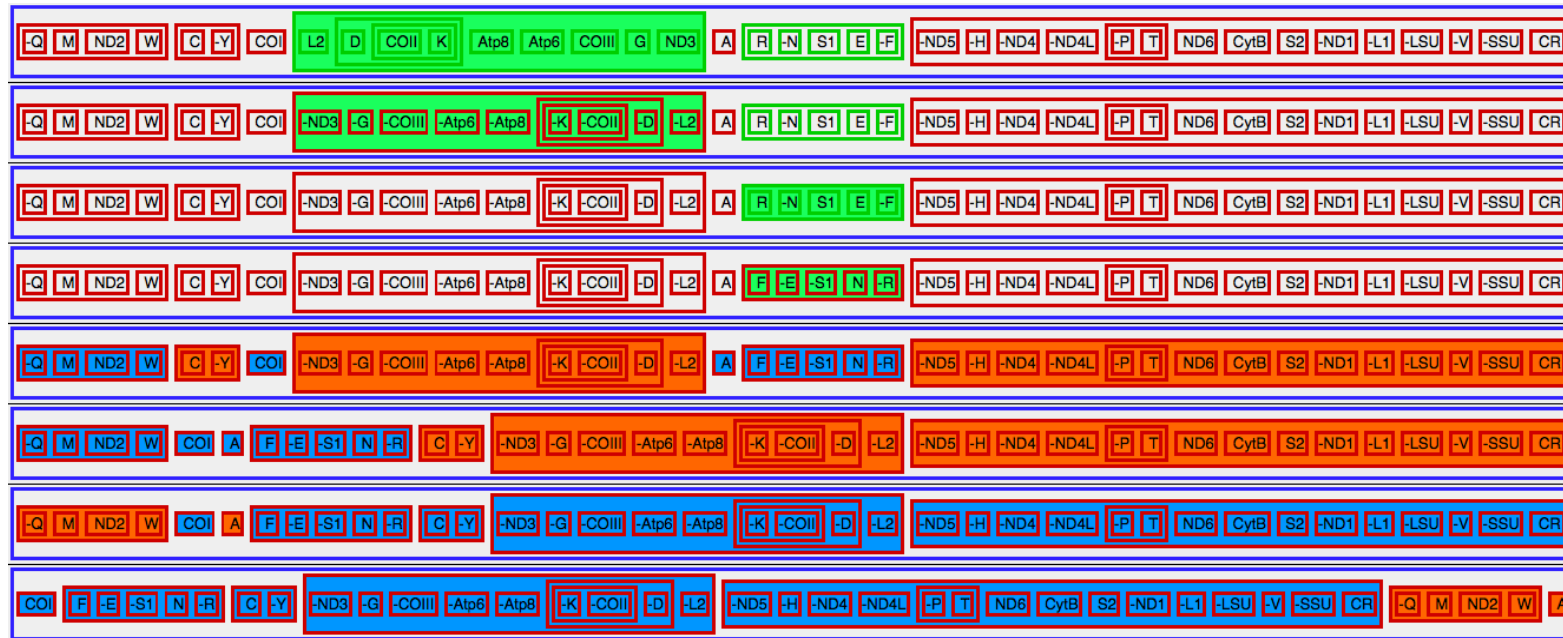

## Reversal

TDRL

TDRL

Supplement: S5 Fig — The two predicted models (I and II) represent two different series of steps inferred using the CREx software. The scenario predicted in Model I require two Reversals, one transposition and one TRDL while Model II shows two Reversals and two TRDLs to arrive at the current mitochondrial gene order found in Orseolia oryzae from the ancestral arthropod gene order. (PDF) [file pone.0134625.s005.pdf]

(a)

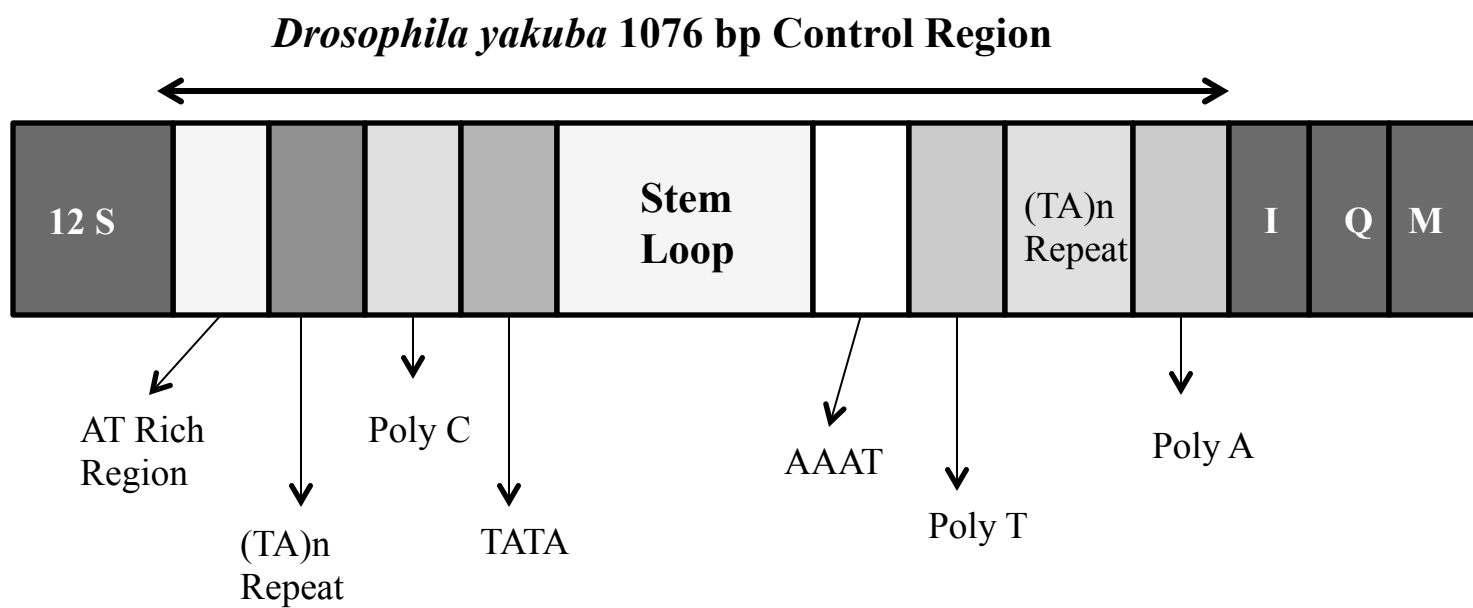

(b)

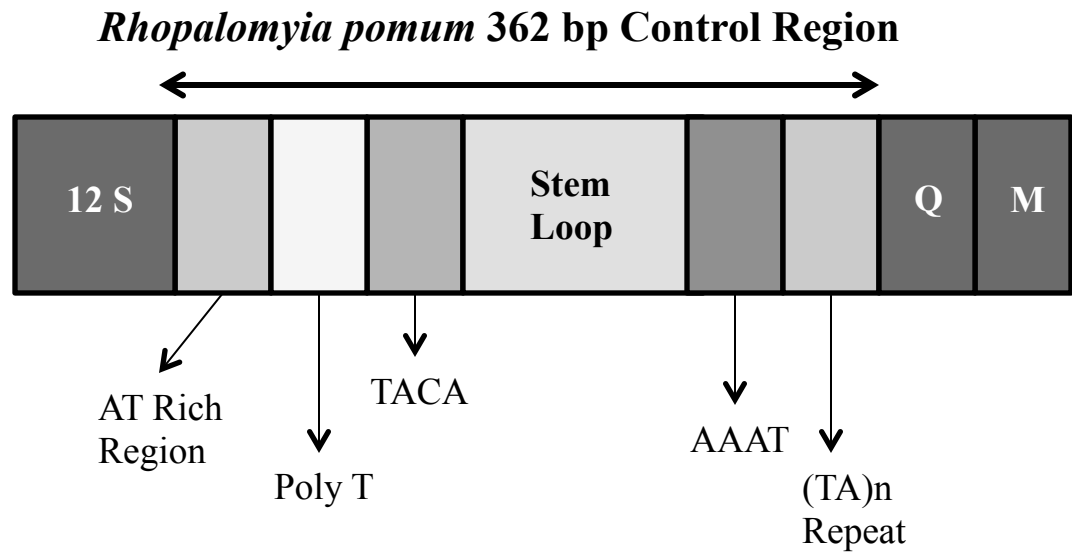

(c)

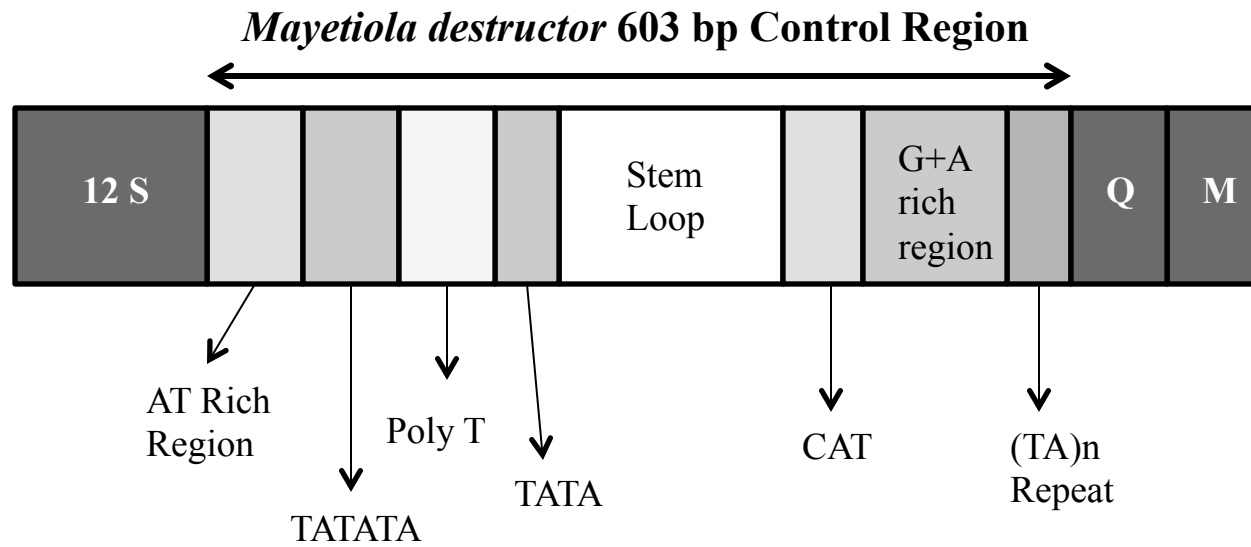

(d)

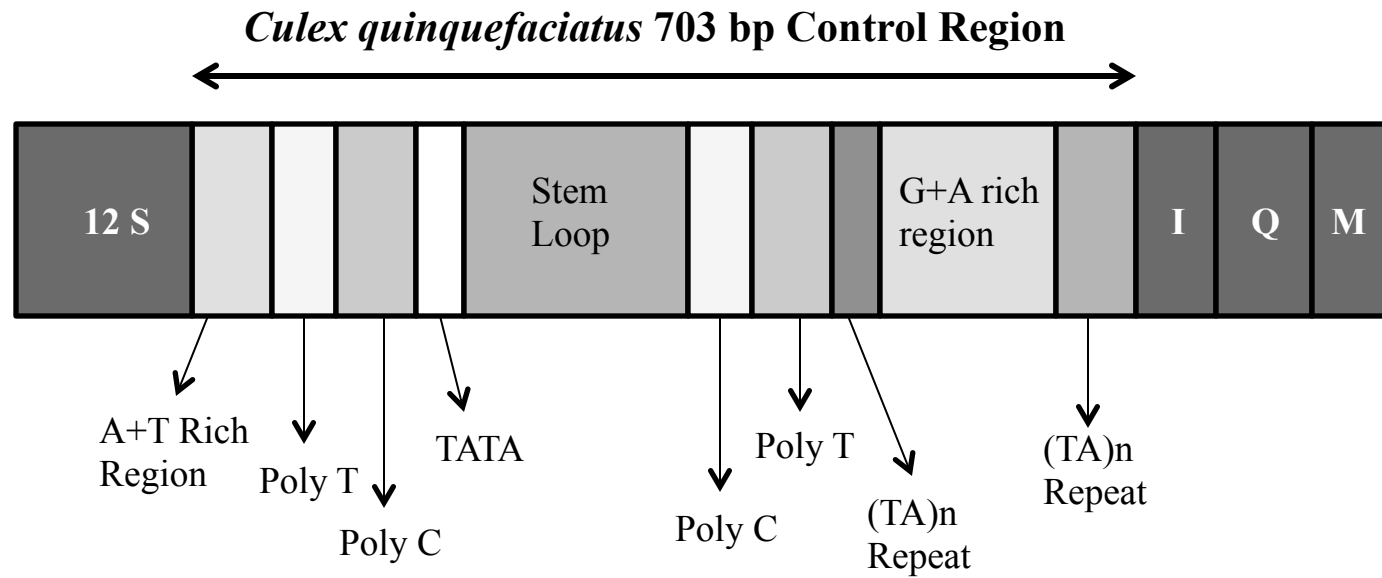

(e)

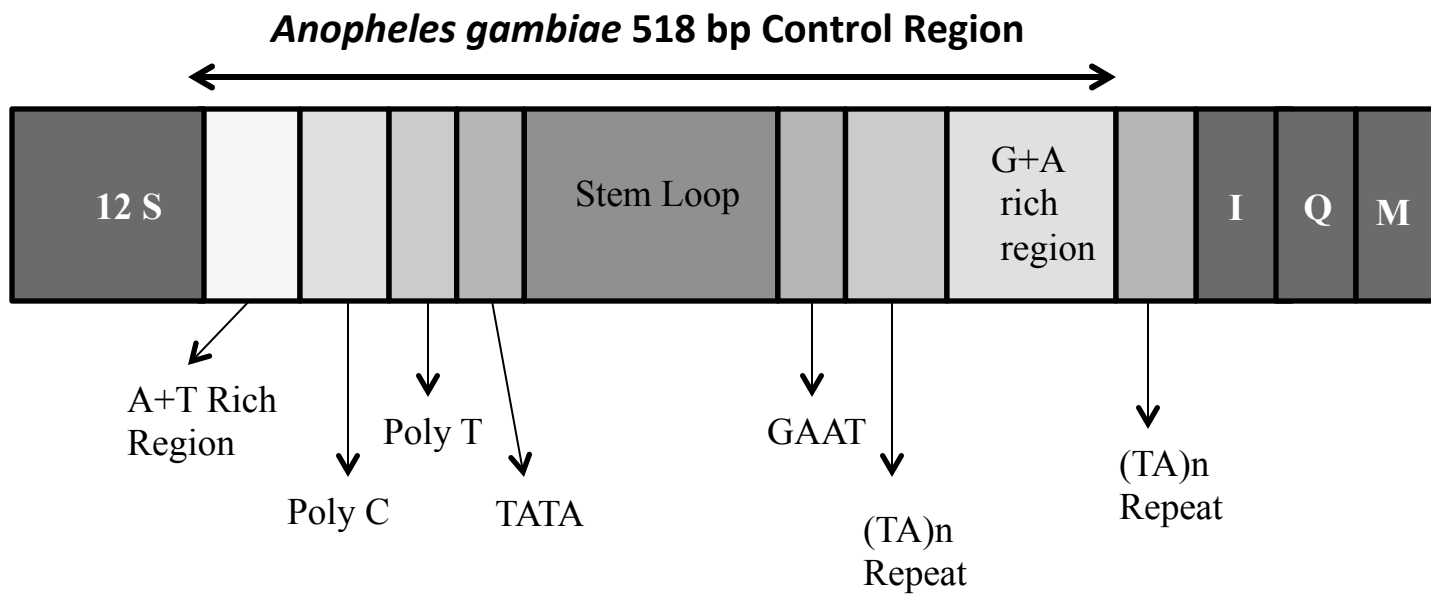

Supplement: S6 Fig — The regions flanking the control region i.e. 12S and trnQ, trnM and ND2 are represented by dark grey boxes (a: Drosophila yakuba, b: Rhopalomyia pomum, c: Mayetiola destructor, d: Culex quinquefasciatus, e: Anopheles gambiae). (PDF) [file pone.0134625.s006.pdf]
