# Supplementary material for: The Asian Rice Gall Midge (Orseolia oryzae) Mitogenome Has Evolved Novel Gene Boundaries and Tandem Repeats That Distinguish Its Biotypes
Source: PLoS One. 2015 Jul 30;10(7):e0134625. doi: 10.1371/journal.pone.0134625 (PMC4520695; doi:10.1371/journal.pone.0134625)
Supplement: S4 Fig — (a) The predicted secondary structure of the putative stem loop region in the control region in different biotypes. (b) Multiple sequence alignment of the putative stem loop region sequences in the control region of different biotypes (GMB1, GMB4, GMB4M and GMB6). Asterisks indicate identical nucleotide residues. (PDF) [file pone.0134625.s004.pdf]

(a)

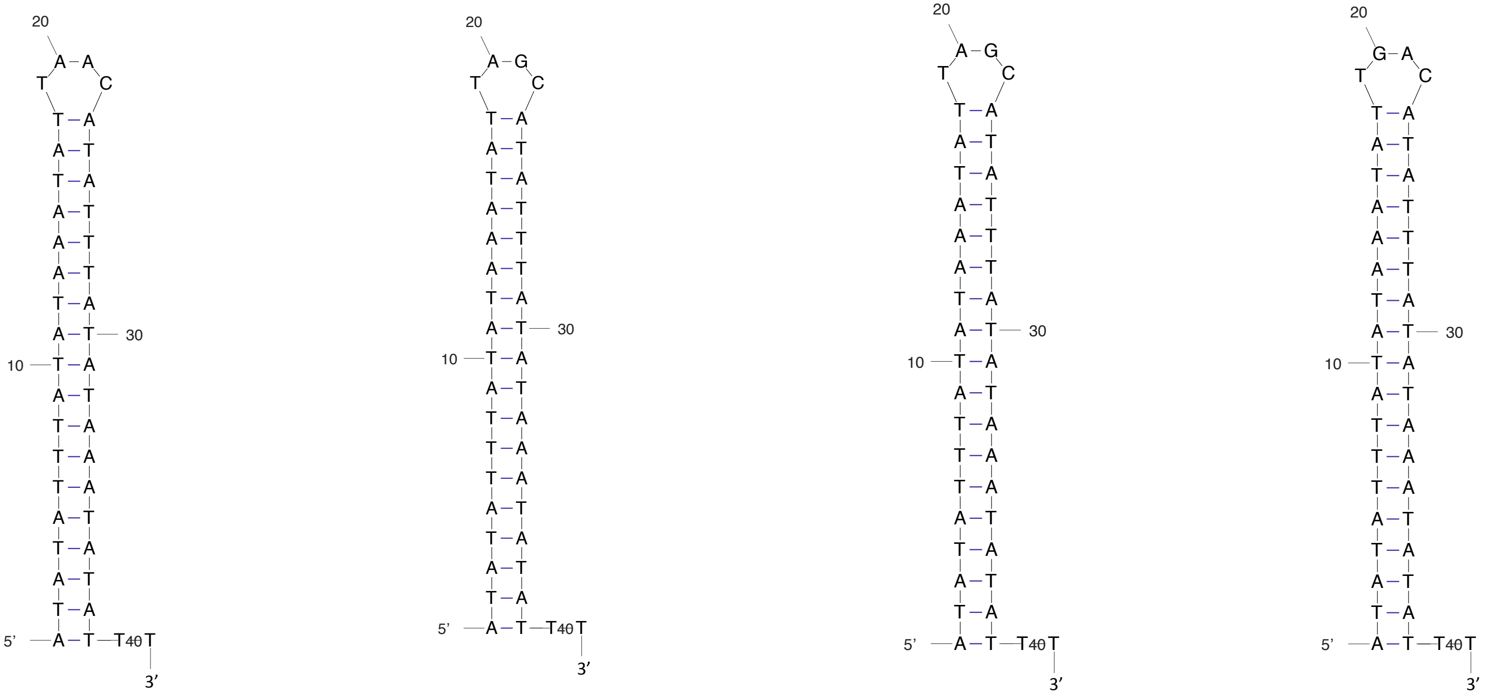

GMB 1 GMB 4 GMB 4M GMB 6

(b)

```
GMB1     ATATATTTATATAAATATTAACATATTTATATAAATATATTT 42
GMB6     ATATATTTATATAAATATTGACATATTTATATAAATATATTT 42
GMB4     ATATATTTATATAAATATTAGCATATTTATATAAATATATTT 42
GMB4M    ATATATTTATATAAATATTAGCATATTTATATAAATATATTT 42
         *******************  *********************
```
