# Supplementary material for: The Asian Rice Gall Midge (Orseolia oryzae) Mitogenome Has Evolved Novel Gene Boundaries and Tandem Repeats That Distinguish Its Biotypes
Source: PLoS One. 2015 Jul 30;10(7):e0134625. doi: 10.1371/journal.pone.0134625 (PMC4520695; doi:10.1371/journal.pone.0134625)
Supplement: S1 File — (PDF) [file pone.0134625.s007.pdf]

S1 File – Supporting Information

Non-coding regions in the gall midge and comparison of different PCG lengths in dipteran mitogenomes

A total of 15 non-coding intergenic spacers were found in the mitogenome of *O. oryzae* accounting for a total of 1,011 bp in the 15,286 bp mitogenome. The longest spacer (155 bp), was located between trn*I* and COI and contained a tandem repeat (with a motif 'ATTTATATTTAA') repeated >5 times. A comparison of the intergenic spacers of *O. oryzae* with intergenic spacers from other insect mitogenomes could not be carried out due to the presence of unique gene boundaries arising due to gene rearrangements in the mitogenome of *O. oryzae*.

In addition, we identified another intergenic spacer containing two penta-nucleotide repeats with the motifs 'TAAAA' and 'AAATT'. The two motifs were iterated 25 and 49 times, respectively and were located between trn*W* and trn*A*. The total percentage of the non-coding region (excluding the control region) was found to be 6.6% that is much higher as compared to other dipterans (Fig 5). The non-coding regions were also screened for the presence of tRNAs. Two trn*L* were detected between trn*I* and COI. Both the tRNAs contained introns in the anti-codon loop region and were predicted to be non-functional copies of the tRNA.

Another feature of the insect mitogenomes is the presence of a 7-bp conserved motif that has been proposed to be the binding site for the mitochondrial termination transcription factor (mtTERM). In the *O. oryzae* mitogenome the mtTERM motif was located between ND1 and trn*L1*. The mitogenome sequences of other insects were also screened for the presence and location of this mtTERM motif. Results (S9 Table) indicated that all three mosquitoes harbour this sequence between trn*S2* and ND1 while it is located between ND4L and trn*T* in *Drosophila yakuba* and between trn*P* and trnL1 in *R. pomum*. However, this motif could not be identified in *M. destructor*.

Comparison of mitochondrial gene sizes across Diptera

	Gene Sizes (bp)													
**Organism**	**ND1**	**ND2**	**ND3**	**ND4**	**ND4l**	**ND5**	**ND6**	**CtyB**	**COI**	**COII**	**COIII**	**ATP6**	**ATP8**	**Control Region**
Orseolia oryzae	903	969	342	1185	300	1698	456	1128	1539	669	780	678	147	588
Mayetiola destructor	905	977	350	1310	269	1674	482	1127	1535	677	782	671	155	603
Rhopalomyia pomum	905	977	350	1296	272	1664	467	1116	1537	674	782	671	155	362
Drosophila yakuba	974	1023	353	1338	290	1719	524	1136	1535	684	788	674	161	1076
Culex quinquefasciatus	956	1022	354	1342	296	1745	515	1134	1536	684	787	680	152	703
Anopheles gambiae	944	1025	353	1341	305	1742	524	1136	1536	684	786	680	161	518
Aedes aegypti	941	1025	328	1343	296	1742	521	1134	1536	684	787	680	161	1882
