# Supplementary material for: The Asian Rice Gall Midge (Orseolia oryzae) Mitogenome Has Evolved Novel Gene Boundaries and Tandem Repeats That Distinguish Its Biotypes
Source: PLoS One. 2015 Jul 30;10(7):e0134625. doi: 10.1371/journal.pone.0134625 (PMC4520695; doi:10.1371/journal.pone.0134625)
Supplement: S2 File — (PDF) [file pone.0134625.s008.pdf]

S2 File – Supporting Information

Truncated tRNAs

Usually in most animals all the mitochondrial tRNAs form a clover leaf structure except for trn*S*, which lacks the D-arm. However, several other tRNAs have been postulated to be missing the T-arm or the D-arm in a number of other animals. For example, maximum amount of truncation has been reported in nematodes from the class Secernentea where all of the 22 mitochondrial tRNAs lack the T-arm or the D-arm[S1]. Also, various parasitic arthropods have unusual secondary structures for the mitochondrial tRNAs. In chigger mite, *Leptotrombidium palladium*, 10 tRNAs have the T-arm missing while 9 others lack the D-arm[S2]. In metastriate ticks and honeybee mite trn*C* lacks the D-arm; while in salmon louse trn*R* and trn*A* lack the D-arm and trn*D* lacks the T-arm[S2]. Similarly, in the other gall midges *M. destructor* and *R. pomum* most of the tRNAs lack the T-arm and the 3' end of the acceptor stem[S3]. However, the cecidomyiid tRNAs showed a high level of sequence similarity in the D and the anticodon stems.

The specific reasons for the truncation in mitochondrial tRNAs are unknown, but it has been hypothesized that these may be due to the evolutionary pressure for mitochondrial genomes to attain smaller size[S4]. Nematode mitochondria have an extra elongation factor (EF-Tu) that has evolved to support interaction with tRNAs lacking the T-arm, which further interacts with the C-terminal domain of the conventional EF-Tu[S5]. Previous studies have shown that some aminoacyl tRNA synthases use the acceptor stem as the major recognition site, while some enzymes also use the D-arm. Hence, if the tRNA has a truncation in the T-arm it is still functional and an amionoacyl moiety could still be added to the shrunken tRNA. It has been shown that the bacterial seryl-tRNA synthetases (SerRS) recognize sites other than the acceptor stem and the anticodon. The bacterial SerRS has the ability to recognize the T-arm and the long variable arms of bacterial trn*S*[S6] thus indicating that truncation in tRNAs need not necessarily inhibit function. Furthermore, it is also known that some tRNAs can be imported from the cytosol into the mitochondria[S7]. This suggests that these imported tRNAs would be functional in place of the otherwise truncated or non-functional copies of the tRNAs encoded by the mitogenome. All these factors support the fact that even with truncated tRNAs the growth and survival of these insects are not impeded.

References

S1. Lavrov DV, Brown WM. *Trichinella spiralis* mtDNA: a nematode mitochondrial genome that encodes a putative ATP8 and normally structured tRNAs and has a gene rearrangement relatable to those of coelomate metazoans. Genetics 2001;157: 621-637.

S2. Shao R, Barker SC. Mitochondrial genomes of parasitic arthropods: implications for studies of population genetics and evolution. Parasitology 2006;134: 153-167.

S3. Beckenbach AT, Joy JB. Evolution of the mitochondrial genomes of gall midges (Diptera: Cecidomyiidae): rearrangement and severe truncation of tRNA genes. Genome Biol Evol. 2009;1: 278-287.

S4. Rand DM. Thermal habit, metabolic rate and the evolution of mitochondrial DNA. Trends Ecol Evol. 1994;9: 125-131.

S5. Watanabe Y, Suematsu T, Ohtsuki T. Losing the stem loop structure from metazoan mitochondrial tRNAs and co-evolution of interacting factors. Front Genet. 2014;1: 109.

S6. Asahara H, Himeno H, Tamura K, Nameki N, Hasegawa T, Shimizu M. *Escherichia coli* seryl-tRNA synthetase recognizes $tRNA^{Ser}$ by its characteristic tertiary structure. J Mol Biol. 1994;236: 738-748.

S7. Rubio MT, Rinehart JJ, Krett B, Duvezin-Caubet S, Reichert AS, Söll D, et al. Mammalian mitochondria have the innate ability to import tRNAs by a mechanism distinct from protein import. Proc Natl Acad Sci. 2008;105: 9186-9191.
