# Supplementary material for: The Asian Rice Gall Midge (Orseolia oryzae) Mitogenome Has Evolved Novel Gene Boundaries and Tandem Repeats That Distinguish Its Biotypes
Source: PLoS One. 2015 Jul 30;10(7):e0134625. doi: 10.1371/journal.pone.0134625 (PMC4520695; doi:10.1371/journal.pone.0134625)
Supplement: S3 File — (PDF) [file pone.0134625.s009.pdf]

S3 File –Supporting Information

Control Region

The control region was also analyzed for the presence of repeat elements. *O. oryzae* (GMB1) had two repeat elements, a 97 bp unit repeated 5.5 times and the other, unique to *O. oryzae*, a [TA] unit repeated 16 times. The first repeat spanned a total of 533 bases in the gall midge control region while the [TA] repeat was present in the 3' region immediately following the first repeat. The *M. destructor* control region revealed the presence of a 79 bp unit repeated 7.7 times, spanning across almost the entire control region of 603 bp. A similar pattern was observed for *R. pomum* where a 96 bp repeat was present 3.2 times covering a major part of the control region. Hence, all the three midges revealed the presence of a repeat unit spanning almost the entire control region.

In comparison with the midges, the mosquitoes had smaller repeat units in the control region. The control region of *A. aegypti* had two repeats and probably the reason why *A. aegypti* has a much longer control region compared with the other two mosquito species. The *D. yakuba* control region had a pattern similar to that observed in the three mosquito species.
