# Supplementary material for: The Asian Rice Gall Midge (Orseolia oryzae) Mitogenome Has Evolved Novel Gene Boundaries and Tandem Repeats That Distinguish Its Biotypes
Source: PLoS One. 2015 Jul 30;10(7):e0134625. doi: 10.1371/journal.pone.0134625 (PMC4520695; doi:10.1371/journal.pone.0134625)
Supplement: S1 Table — (PDF) [file pone.0134625.s010.pdf]

S1 Table. List of primers used to amplify the 18 overlapping fragments in the *Orseolia oryzae* mitogenome

S.No.	Primer Used	Sequence	Reference
1.	C1-J1709	AATTGGWGGWTTYGGAAAYTG	[16]
2.	C1-J2195	TGATTCTTTGGWCACCCWGAAGT	[16]
3.	C1-N2776	GGTAATCAGAGTATCGWCGNGG	[16]
4.	C2-J3399	TACTCATARGATCARTATCAYTG	[16]
5.	C2-N3665	CCACAAATTTCTGAACATTG	[16]
6.	C3-J5470	GCAGCTGCYTGATAYTGRCA	[16]
7.	CB-J10933	GTTCTACCTTGAGGNCAAATRTC	[16]
8.	CB-N11010	TATCTACAGCRAATCCYCCYCA	[16]
9.	LR-J12888	CCGGTTTGAACTCARATCATGTAA	[16]
10.	LR-N13000	TTACCTTAGGGATAACAGCGTAA	[16]
11.	N1-J11876	CGAGGTAAAGTMCCWCGAACYCA	[16]
12.	N1-J12261	AACTTCATAAGAAATAGTYTGRGC	[16]
13.	N1-N12067	AATCGWACTCCWTTTGATTTTGC	[16]
14.	N1-N12595	GTWGCTTTTTTAACTTTATTRGARCG	[16]
15.	N2-J586	CCATTTCAYTTYTGATTYCC	[16]
16.	N2-N993	GGTAAAAATCCTAAAAATGGNGG	[16]
17.	N4-J8641	CCAGAAGAACATAANCCRTG	[16]
18.	N4-L9629	GTTTGTGAGGGWGYTTTRGG	[16]
19.	N4L-J9648	ACCTAAAGCTCCCTCACAWAC	[16]
20.	N4-N8727	AAATCTTTRATTGCTTATTCWTC	[16]
21.	N5-J7572	AAAGGGAATTTGAGCTCTTTTWGT	[16]
22.	SR-J14197	GTACAYCTACTATGTTACGACTT	[16]
23.	SR-N14745	GTGCCAGCAGYYGCGGTTANAC	[16]
24.	Cox2-Atp6Cox3 F	AAGTAGATGCAATTCCAGGTCGT	Present Study
25.	Cox2-Atp6Cox3 R	AATAGATCCAATCAAAGGTCAAGGACT	Present Study
26.	HN F	TACCATCAATACCTCAATAAGT	Present Study
27.	HN R	ATCCCCCAATTATTACTAAAAA	Present Study
28.	NC F	AGGATTACCTCCATTTTTAG	Present Study
29.	NC R	ACCTGTATGAGCAATAGAAG	Present Study
30.	CN2 F	AAGGTTTTTTCATTTTAGTTTATG	Present Study
31.	CN2 R	GAGATGGTTTAGGATTAGTATC	Present Study
32.	RN1 F	AAAATAATAGGGTATCTAATCC	Present Study
33.	RN1 R	GGAAATCAAAAATGAAATGG	Present Study
34.	CC R	CCCTAAAGAAGGAATAGTTC	Present Study
35.	35 R	TATTTAAGGCTTATTATTTCTTTGTGC	Present Study
