# Supplementary material for: The Asian Rice Gall Midge (Orseolia oryzae) Mitogenome Has Evolved Novel Gene Boundaries and Tandem Repeats That Distinguish Its Biotypes
Source: PLoS One. 2015 Jul 30;10(7):e0134625. doi: 10.1371/journal.pone.0134625 (PMC4520695; doi:10.1371/journal.pone.0134625)
Supplement: S2 Table — (PDF) [file pone.0134625.s011.pdf]

S2 Table. Accession numbers of all the species used for comparison in the present study

Species	GenBank Accession numbers
Abispa_ephippium	NC_011520
Aedes aegypti	NC_010241
Anopheles gambiae	NC_002084
Apis mellifera	NC_001566
Bothriometopus macrocnemis	NC_009983
Campanulotes bidentatus compar	NC_007884
Culex quinquefasciatus	NC_014574
Delias hyparete	NC_020428
Diadegma semiclausum	NC_012708
Diuraphis noxia	NC_022727
Drosophila yakuba	NC_001322
Helicoverpa armigera	NC_014668
Heterodoxus macropus	NC_002651
Laodelphax striatella	JX880068
Locusta migratoria	NC_001712
Mayetiola destructor	GQ387648
Nilaparvata lugens	NC_021748
Orseolia oryzae	KM888183
Panonychus citri	HM189212
Rhopalomyia pomum	GQ387649
Schistocerca gregaria gregaria	NC_013240
Schizaphis graminum	NC_006158
Tetranychus urticae	NC_010526
Thrips imaginis	NC_004371
Tribolium castaneum	AJ312413
Vanhornia eucnemidarum	NC_008323
