# Supplementary material for: The Asian Rice Gall Midge (Orseolia oryzae) Mitogenome Has Evolved Novel Gene Boundaries and Tandem Repeats That Distinguish Its Biotypes
Source: PLoS One. 2015 Jul 30;10(7):e0134625. doi: 10.1371/journal.pone.0134625 (PMC4520695; doi:10.1371/journal.pone.0134625)
Supplement: S3 Table — (PDF) [file pone.0134625.s012.pdf]

S3 Table. A comparison of start and stop codons for PCGs across different species in Diptera

Gene	Start Codon							Stop Codon						
	O. oryzae	*M. destructor*	*R. pomum*	*D. yakuba*	*C. quinquefasciatus*	*A. gambiae*	*A. aegypti*	*O. oryzae*	*M. destructor*	*R. pomum*	*D. yakuba*	*C. quinquefasciatus*	*A. gambiae*	*A. aegypti*
Atp6	ATG	ATG	ATG	ATG	ATG	ATG	ATG	TAA	TAA	TAA	TAA	TAA	TAA	TAA
Atp8	ATA	ATT	ATT	ATT	ATA	ATC	ATT	TAA	TAA	TAA	TAA	TAA	TAA	TAA
COI	ATT	ATT	N.A.	TCG	N.A.	N.A.	TCG	TAA	TAA	TA-	TAA	T--	T--	T--
COII	ATT	ATA	ATA	ATG	ATG	ATG	ATG	TAA	TAA	TAA	T--	T--	T--	T--
COIII	ATT	ATA	TTA	ATG	ATG	ATG	ATG	TAA	TAA	TAA	TAA	TA-	T--	TA-
CytB	ATT	ATT	ATA	ATG	ATG	ATG	ATG	TAA	TAA	T--	TAA	T--	TAA	T--
ND1	ATA	ATG	ATA	ATA	ATA	ATA	ATA	TAA	TAA	TAA	TAA	TAA	TAA	TAA
ND2	ATT	ATA	ATA	ATT	ATC	ATC	ATT	TAA	TAA	TAA	T--	TAA	TAA	TAA
ND3	ATT	ATT	ATA	ATT	ATA	ATA	ATT	TAA	TAA	TAA	TAA	T--	TAA	TA-
ND4	ATA	ATT	ATA	ATG	ATG	ATG	ATG	TAA	TAG	T--	T--	TA-	T--	TAA
ND4L	ATT	TTG	ATA	ATG	ATG	ATA	ATG	TAA	TAA	TAA	TAA	TAA	TAA	TAA
ND5	ATA	ATT	ATT	ATT	ATC	ATG	ATC	TAA	TAA	TAA	T--	TAA	TAA	TAA
ND6	ATA	ATG	ATA	ATT	ATA	ATT	ATT	TAG	TAA	TAA	TAA	TAA	TAA	TAA

Note: Accession numbers of the mitogenomes used in this comparison are provided in S2 Table
