# Supplementary material for: The Asian Rice Gall Midge (Orseolia oryzae) Mitogenome Has Evolved Novel Gene Boundaries and Tandem Repeats That Distinguish Its Biotypes
Source: PLoS One. 2015 Jul 30;10(7):e0134625. doi: 10.1371/journal.pone.0134625 (PMC4520695; doi:10.1371/journal.pone.0134625)
Supplement: S4 Table — (PDF) [file pone.0134625.s013.pdf]

S4 Table. Distribution of PCGs and tRNAs on the major (J) and minor (N) strands across different species in Diptera

Organism	J-Strand		N-Strand	
	tRNAs	tRNAs	PCGs	
O. oryzae	14	8	4	9
M. destructor	12	10	4	9
R. pomum	12	10	4	9
A. gambiae	13	9	4	9
C. quinquefasciatus	14	8	4	9
A. aegypti	14	8	4	9
D. yakuba	15	7	4	9

Note: Accession numbers of the mitogenomes used in this comparison are mentioned in the S2 Table
