# Supplementary material for: The Asian Rice Gall Midge (Orseolia oryzae) Mitogenome Has Evolved Novel Gene Boundaries and Tandem Repeats That Distinguish Its Biotypes
Source: PLoS One. 2015 Jul 30;10(7):e0134625. doi: 10.1371/journal.pone.0134625 (PMC4520695; doi:10.1371/journal.pone.0134625)
Supplement: S5 Table — (PDF) [file pone.0134625.s014.pdf]

S5 Table. Composition of the control region across Diptera

Organism	Length	A+T %	5' Consensus	3' Consensus	No. of $(TA)_n$ Blocks	AT rich domain	polyT	polyC	polyA	polyG
Orseolia oryzae	578	93.8	TATA	GAAT	1	96.2	5'	-	-	-
Mayetiola destructor	603	90.9	TATA	CAT	1	96.0	5'	-	-	-
Rhopalomyia pomum	362	94.2	TACA	AAAT	1	95.4	5'	-	-	-
Drosophila yakuba	1076	92.9	TATA	AAAT	2	96.5	3'	+	+	-
Anopheles gambiae	518	94.2	TATA	GAAT	2	99.0	5'	+	+	-
Culex quinquefasciatus	703	88.5	TATA	×	2	96.0	5'	+	+	-
Aedes aegypti	2070	93.1	TATA	GAT	2	97.8	5'	-	+	+

(+: present, -: absent)

Note: Accession numbers of the mitogenomes used in this comparison are mentioned in the S2 Table
