# Supplementary material for: The Asian Rice Gall Midge (Orseolia oryzae) Mitogenome Has Evolved Novel Gene Boundaries and Tandem Repeats That Distinguish Its Biotypes
Source: PLoS One. 2015 Jul 30;10(7):e0134625. doi: 10.1371/journal.pone.0134625 (PMC4520695; doi:10.1371/journal.pone.0134625)
Supplement: S6 Table — (PDF) [file pone.0134625.s015.pdf]

S6 Table. A Comparison of the repeats present in control region of different species of Diptera

Species	Length of the Motif (bp)	No. of Repeats
O. oryzae	97	5.5
M. destructor	79	7.7
R. pomum	96	3.2
D. yakuba	19	3.1
A. gambiae	21	3
C. quinquefasciatus	18	3.6
A. aegypti	43	4.1

Note: Accession numbers of the mitogenomes used in this comparison are mentioned in S2 Table
