# Supplementary material for: The Asian Rice Gall Midge (Orseolia oryzae) Mitogenome Has Evolved Novel Gene Boundaries and Tandem Repeats That Distinguish Its Biotypes
Source: PLoS One. 2015 Jul 30;10(7):e0134625. doi: 10.1371/journal.pone.0134625 (PMC4520695; doi:10.1371/journal.pone.0134625)
Supplement: S7 Table — (PDF) [file pone.0134625.s016.pdf]

S7 Table. Number of repeats present in the control region of different biotypes of *Orseolia oryzae*

Biotype	Size (bp) of Control region	Repeat I		Repeat II	
		Length of motif	No. of repeats	Length of motif	No. of repeats
**GMB 1**	578	TA	16	97	5.5
**GMB 4**	582	TA	12	103	5.5
**GMB 4M**	608	TA	4	97	6.1
**GMB 6**	586	TA	17	97	5.5

Note: The control region could not be amplified from the remaining biotypes
