# Supplementary material for: The Asian Rice Gall Midge (Orseolia oryzae) Mitogenome Has Evolved Novel Gene Boundaries and Tandem Repeats That Distinguish Its Biotypes
Source: PLoS One. 2015 Jul 30;10(7):e0134625. doi: 10.1371/journal.pone.0134625 (PMC4520695; doi:10.1371/journal.pone.0134625)
Supplement: S8 Table — (PDF) [file pone.0134625.s017.pdf]

S8 Table. Organization of the control region in different biotypes of *Orseolia oryzae*

Biotype	Length (bp)	A+T %	5' consensus	3' consensus	[TA]n Repeats	poly T
**GMB 1**	578	93.8	TATA	GAAT	16	5'
**GMB 4**	582	93.6	TATA	GAAT	12	5'
**GMB 4M**	608	93.1	TATA	GAAT	4	5'
**GMB 6**	586	93.7	TATA	GAAT	17	5'

Note: The control region could not be amplified from the remaining biotypes
