# Supplementary material for: The Asian Rice Gall Midge (Orseolia oryzae) Mitogenome Has Evolved Novel Gene Boundaries and Tandem Repeats That Distinguish Its Biotypes
Source: PLoS One. 2015 Jul 30;10(7):e0134625. doi: 10.1371/journal.pone.0134625 (PMC4520695; doi:10.1371/journal.pone.0134625)
Supplement: S9 Table — (PDF) [file pone.0134625.s018.pdf]

S9 Table. The location of the mtTERM sequence across different species in Diptera

Organism	Location	Strand
Orseolia oryzae	ND1-*trnL1*	Major
Mayetiola destructor	Absent	N.A.
Rhopalomyia pomum	*trnP-trnI*	Major
Drosophila yakuba	ND4L-*trnT*	Minor
Culex quinquefasciatus	*trnS2*-ND1	Major
Anopheles gambiae	*trnS2*-ND1	Major
Aedes aegypti	*trn S2*-ND1	Major

Note: Accession numbers of the mitogenomes used in this comparison are mentioned in the S2 Table
